# Alkaline-adaptive covalent organic framework photocatalysts: synergistic molecular orbital and hydrogen-bond network engineering for H_2_O_2_ production

**DOI:** 10.1039/d5sc08298f

**Published:** 2026-01-29

**Authors:** Zhiwu Yu, Jiayi Zhang, Xiaolong Zhang, Xuwen Sun, Guihong Wu, Zhiyun Zhang, Fengtao Yu, Jianli Hua

**Affiliations:** a Key Laboratory for Advanced Materials, Joint International Research Laboratory for Precision Chemistry and Molecular Engineering, Feringa Nobel Prize Scientist Joint Research Center, Frontiers Science Center for Materiobiology and Dynamic Chemistry, School of Chemistry and Molecular Engineering, East China University of Science and Technology Shanghai 200237 China jlhua@ecust.edu.cn; b Jiangxi Province Key Laboratory of Functional Organic Polymers, East China University of Technology Nanchang 330013 China fty853815622@ecut.edu.cn

## Abstract

Alkaline hydrogen peroxide (H_2_O_2_) is highly desirable for critical applications due to its superior stability and reactivity, but it is incompatible with conventional near-neutral production methods. While covalent organic frameworks (COFs) show promise for photocatalytic H_2_O_2_ generation, their alkaline performance is severely limited by poor charge dynamics and inadequate hydrophilicity, hindering the essential 2e^−^ oxygen reduction reaction (ORR: O_2_ + 2e^−^ + H_2_O → HO_2_^−^ + OH^−^) and 4e^−^ water oxidation reaction (WOR: 4OH^−^ → O_2_ + 2H_2_O + 4e^−^). This work pioneers a dual-engineering strategy (molecular orbital and interfacial hydrogen-bonding network engineering) within β-ketoenamine-linked COFs to overcome these challenges simultaneously. By contrasting phenazine-based (TP-PZ-COF) and anthracene-based (TP-AN-COF) COFs, we demonstrate that strategic integration of sp^2^-N heteroatoms modulates molecular orbitals and enhances n → π* transitions, optimizing charge separation and transport for efficient 2e^−^ ORR and 4e^−^ WOR. Concurrently, the planar phenazine units form robust hydrogen-bonding networks that dramatically boost hydroxide ion (OH^−^) affinity and interfacial enrichment, thereby accelerating the 4e^−^ WOR kinetics. This integrated approach enabled TP-PZ-COF to achieve an exceptional alkaline H_2_O_2_ production rate of 4961 µmol g^−1^ h^−1^ in 0.01 M NaOH, representing an 8.1-fold increase over TP-AN-COF (606 µmol g^−1^ h^−1^). The generated H_2_O_2_ efficiently degraded industrial dye pollutants. Direct experimental and theoretical validations confirmed the cooperative mechanism between charge dynamics optimization and OH^−^ affinity enhancement, providing a new blueprint for designing on-demand alkaline H_2_O_2_ photocatalysts.

## Abstract 2

A phenazine-based COF was engineered *via* molecular-orbital modulation and hydrogen-bond network construction, improving charge separation/transfer and OH^−^ adsorption for efficient alkaline H_2_O_2_ production *via* the cooperative 2e^−^ ORR/4e^−^ WOR.

## Graphical abstract

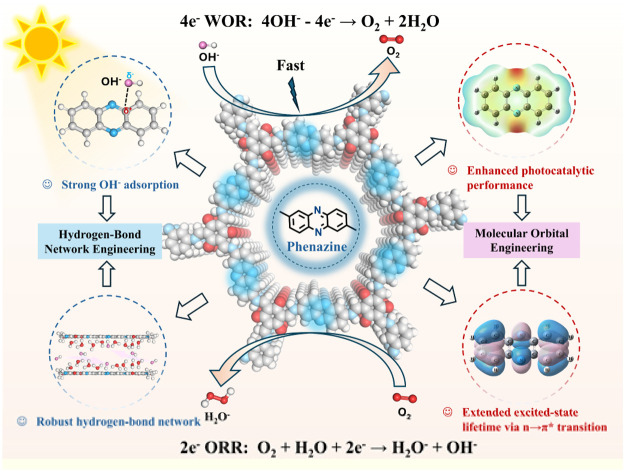

## Introduction

Hydrogen peroxide, a green oxidant decomposing solely to water and oxygen, is essential for applications like pulp bleaching,^1^ disinfection,^2^ organic synthesis,^3^ and environmental remediation.^4^ Its utilization under alkaline conditions is particularly advantageous, enhancing stability and facilitating the generation of highly reactive hydroxyl radicals (˙OH), thereby boosting efficiency in advanced oxidation processes, selective transformations, and electrochemical sensing.^5–7^ However, conventional H_2_O_2_ production relies heavily on the energy-intensive anthraquinone process, which inherently yields near-neutral H_2_O_2_.^8,9^ This fundamental limitation prevents the immediate exploitation of alkaline H_2_O_2_ superior reactivity and stability in distributed applications.^10^ Consequently, developing efficient photocatalytic systems for direct alkaline H_2_O_2_ generation is not just beneficial but urgently necessary for sustainable, on-site production in its most effective form.

COFs have rapidly emerged as premier photocatalysts for H_2_O_2_ production due to their crystalline order, exceptional tunability, photostability, and abundant functional sites.^11–13^ Unlike traditional inorganic semiconductors, COFs offer unparalleled molecular-level control over the electronic structure and active sites through rational backbone and linker design.^14–17^ This enables precise optimization of light harvesting, charge carrier generation, and catalytic activity specifically for H_2_O_2_ synthesis.^18–21^ Nevertheless, deploying COFs under desirable alkaline conditions presents significant hurdles: (i) inefficient charge separation and transfer kinetics, which severely hinder the critical two-electron oxygen reduction reaction (2e^−^ ORR) pathway; (ii) often insufficient hydrophilicity, which critically limits the adsorption of OH^−^ and consequently hampers the formation of efficient reactant interfaces, drastically retarding the 4e^−^ WOR kinetics; and (iii) inadequate chemical and structural stability in strongly basic environments, where framework degradation or active-site deactivation often leads to rapid performance decay during photocatalysis. These combined limitations fundamentally bottleneck the overall photocatalytic H_2_O_2_ yield in alkaline media. Building upon the identified challenges in alkaline environments, modulating molecular orbitals and constructing hydrogen-bonding networks within COFs emerge as particularly promising strategies to overcome these bottlenecks and enhance photocatalytic H_2_O_2_ production.^22–25^ Molecular orbital engineering, achieved through heteroatom incorporation (*e.g.*, N, S, and O) into the COF backbone, directly targets electronic structure and energy level optimization.^26,27^ This approach holds significant potential to address inefficient charge separation and transfer, which are key factors currently limiting the 2e^−^ ORR pathway, thereby improving light harvesting, promoting exciton dissociation, and facilitating charge carrier transport. Simultaneously, the strategic introduction of robust hydrogen-bonding networks offers a potent solution to the critical issue of insufficient hydrophilicity under alkaline conditions.^28^ These networks enhance water/ion affinity, promote the targeted enrichment of reactive OH^−^, accelerate intermediate conversion, and reduce energy barriers, effectively mitigating the slow 4e^−^ WOR kinetics caused by poor OH^−^ adsorption and interfacial inefficiency.^29,30^ While existing phenazine-COFs and β-ketoenamine COFs can address certain individual limitations, they still suffer from single, isolated shortcomings under strongly alkaline conditions, such as limited charge separation, insufficient OH^−^ adsorption, or low H_2_O_2_ stability, which restricts their overall photocatalytic performance. While each strategy individually tackles specific bottlenecks, the synergistic integration of molecular orbital engineering and hydrogen-bonding network construction within a single COF system remains largely unexplored but highly promising for efficient alkaline photocatalytic H_2_O_2_ generation.

**Fig. 1:**
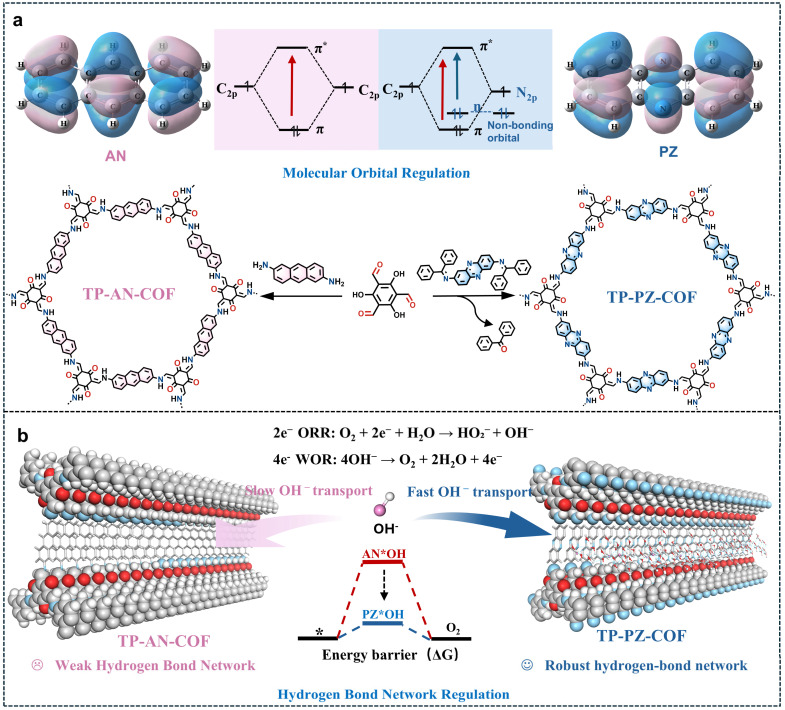
(a) Synthetic routes and corresponding molecular orbitals of TP-PZ-COF and TP-AN-COF. (b) Engineering of hydrogen-bonding networks.

Herein, we developed a dual-engineering strategy integrating both approaches within β-ketoenamine-linked COFs. Two structurally analogous COFs TP-AN-COF and TP-PZ-COF, which were different only in two linker atoms, were synthesized for direct performance evaluation (Fig. 1a). The phenazine-based TP-PZ-COF demonstrated substantially enhanced photocatalytic activity, attributed synergistically to (i) expanded n → π* excitation channels optimizing charge dynamics, and (ii) planar sp^2^-hybridized nitrogen atoms establishing robust hydrogen bonds with H_2_O/OH^−^ (Fig. 1b). Beyond improving reaction kinetics, these hydrogen-bonding interactions help stabilize the local chemical environment of the framework, thereby enhancing the structural durability of the COF in strongly alkaline media. This dual functionality collectively addressed prior bottlenecks, enhancing charge separation for efficient 2e^−^ ORR while accelerating OH^−^ enrichment and activation for improved WOR kinetics. Under alkaline conditions (0.01 M NaOH), TP-PZ-COF achieved an exceptional H_2_O_2_ production rate of 4961 µmol g^−1^ h^−1^, surpassing TP-AN-COF by 8.1-fold. The practical utility of this alkaline-generated H_2_O_2_ was further demonstrated through effective degradation of metal-containing dyes (*e.g.*, rhodamine B and rose bengal) in industrial wastewater. Transient absorption spectroscopy (TAS) and density functional theory (DFT) calculations unambiguously revealed the cooperative mechanism: molecular orbital modulation directs charge transfer pathways, while hydrogen-bond networks facilitate reactant interfacial dynamics. This work establishes a new design paradigm for COF photocatalysts targeting efficient on-demand H_2_O_2_ synthesis in alkaline environments.

## Results and discussion

**Fig. 2:**
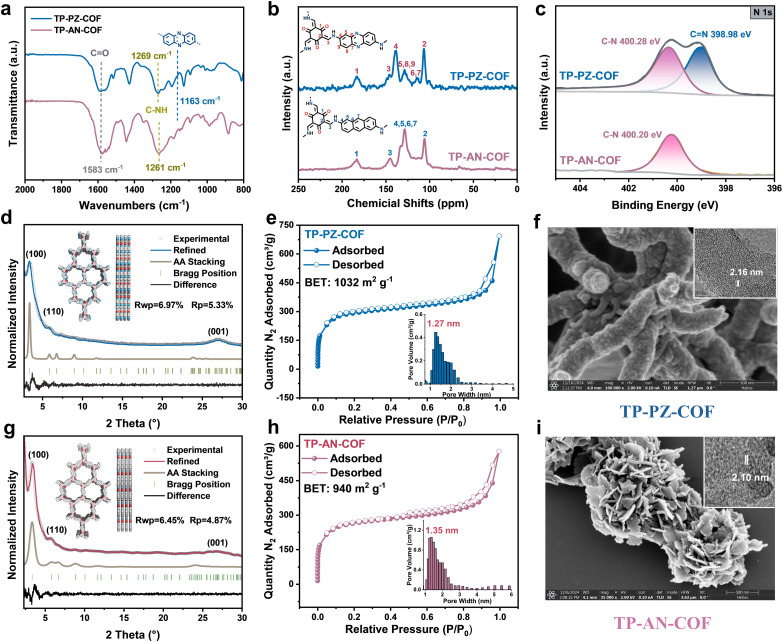
(a) FT-IR spectrum of TP-PZ-COF and TP-AN-COF. (b) Solid-state ^13^C CP/MAS-NMR spectra of TP-PZ-COF and TP-AN-COF. (c) The XPS spectra of N 1s for TP-PZ-COF and TP-AN-COF. (d) PXRD patterns of TP-PZ-COF. (e) N_2_ adsorption–desorption isotherms at 77.3 K and pore size distribution (inset) for TP-PZ-COF. (f) SEM and HR-TEM image (inset) of TP-PZ-COF. (g) PXRD patterns of TP-AN-COF. (h) N_2_ adsorption–desorption isotherms at 77.3 K and pore size distribution (inset) for TP-AN-COF. (i) SEM and HR-TEM image (inset) of TP-PZ-COF.

TP-AN-COF was synthesized by a typical Schiff-base condensation reaction between 1,3,5-triformylphloroglucinol (TFP) and 2,6-anthracenediamine, while TP-PZ-COF was obtained *via* an imine-exchange reaction between TFP and 2,7-diaminophenazine·benzylideneaniline (DAPH·Bnzph).^31^ The chemical structures of both COFs were systematically characterized using Fourier-transform infrared (FT-IR) spectroscopy, solid-state ^13^C nuclear magnetic resonance (^13^C NMR), and X-ray photoelectron spectroscopy (XPS). The formation of β-ketoenamine linkages in both frameworks was confirmed by FT-IR analysis (Fig. S2). Specifically, characteristic stretching bands corresponding to C=O appeared at ∼1583 cm^−1^, while C–N stretching vibrations were observed at 1261 and 1269 cm^−1^. Additionally, a distinct absorption peak at ∼1163 cm^−1^ was assigned to the phenazine (PZ) moiety (Fig. 2a).^32^ Solid-state ^13^C NMR spectra further corroborated the formation of β-ketoenamine linkages, showing signals at ∼183 ppm for C=O, ∼105 ppm for C=C–NH, and ∼145 ppm for =C–NH carbons (Fig. 2b). High-resolution XPS analysis provided additional evidence for the successful construction of the two COF structures (Fig. 2c and S4). For TP-AN-COF, the N 1s spectrum showed a single deconvoluted peak at 400.28 eV, corresponding to sp^3^-hybridized N in C–N bonds of the β-ketoenamine linkage. In contrast, TP-PZ-COF displayed a new peak at 398.98 eV, attributable to sp^2^-hybridized N (C=N), confirming the successful incorporation of the PZ units into the COF. The crystalline structures of TP-PZ-COF and TP-AN-COF were investigated by powder X-ray diffraction (PXRD), as shown in Fig. 2d and g. TP-PZ-COF exhibited distinct diffraction peaks at 3.38°, 5.86°, and 23.8°, corresponding to the (100), (110), and (001) crystal planes, respectively. Similarly, TP-AN-COF displayed diffraction peaks at 3.34°, 5.79°, and 23.8°, assignable to the (100), (110), and (001) facets. Both COFs presented comparable crystalline domain sizes, as summarized in Tables S1 and S2. The experimental PXRD patterns were in excellent agreement with simulated diffraction profiles based on an AA stacking model (Fig. S5 and S6). The refined unit cell parameters were as follows: TP-PZ-COF, *a* = *b* = 30.15 Å, *c* = 3.74 Å, *α* = *β* = 90.00°, *γ* = 120.00°, with residuals *R*_wp_ = 6.97% and *R*_p_ = 5.33%; TP-AN-COF, *a* = *b* = 30.48 Å, *c* = 3.72 Å, *α* = *β* = 90.00°, *γ* = 120.00°, with residuals *R*_wp_ = 6.45% and *R*_p_ = 4.87%. These results clearly confirm that both COFs possess highly ordered crystalline structures with well-defined, predesigned topologies.^33^

The porous structures of TP-PZ-COF and TP-AN-COF were investigated by N_2_ adsorption–desorption measurements. As shown in Fig. 2e and h, both COFs exhibit typical type-I isotherms, indicative of microporous structures. The Brunauer–Emmett–Teller (BET) surface areas were determined to be 1032 m^2^ g^−1^ for TP-PZ-COF and 940 m^2^ g^−1^ for TP-AN-COF, providing a comparable basis for evaluating their photocatalytic performance. Pore size distribution analyses based on nonlocal density functional theory (NLDFT) with a cylindrical pore model revealed distinct peaks centered at 1.27 and 1.35 nm for TP-PZ-COF and TP-AN-COF. Such microporous architectures are favorable for constructing hydrogen-bonded networks and facilitating the diffusion of O_2_ and OH^−^ ions, thereby enhancing photocatalytic H_2_O_2_ generation under alkaline conditions.^34^ Scanning electron microscopy (SEM) images revealed that TP-PZ-COF adopted a partially flexible rod-like morphology (Fig. 2f and S7), while TP-AN-COF exhibited a sheet-like morphology (Fig. 2i and S8), suggesting that the PZ unit confers greater structural flexibility than the anthracene (AN) counterpart. High-resolution transmission electron microscopy (HR-TEM) confirmed the high crystallinity of both COFs, with well-resolved lattice fringes corresponding to interplanar spacings of 2.16 and 2.10 nm for TP-PZ-COF and TP-AN-COF (insets in Fig. 2f and i).^35^

**Fig. 3:**
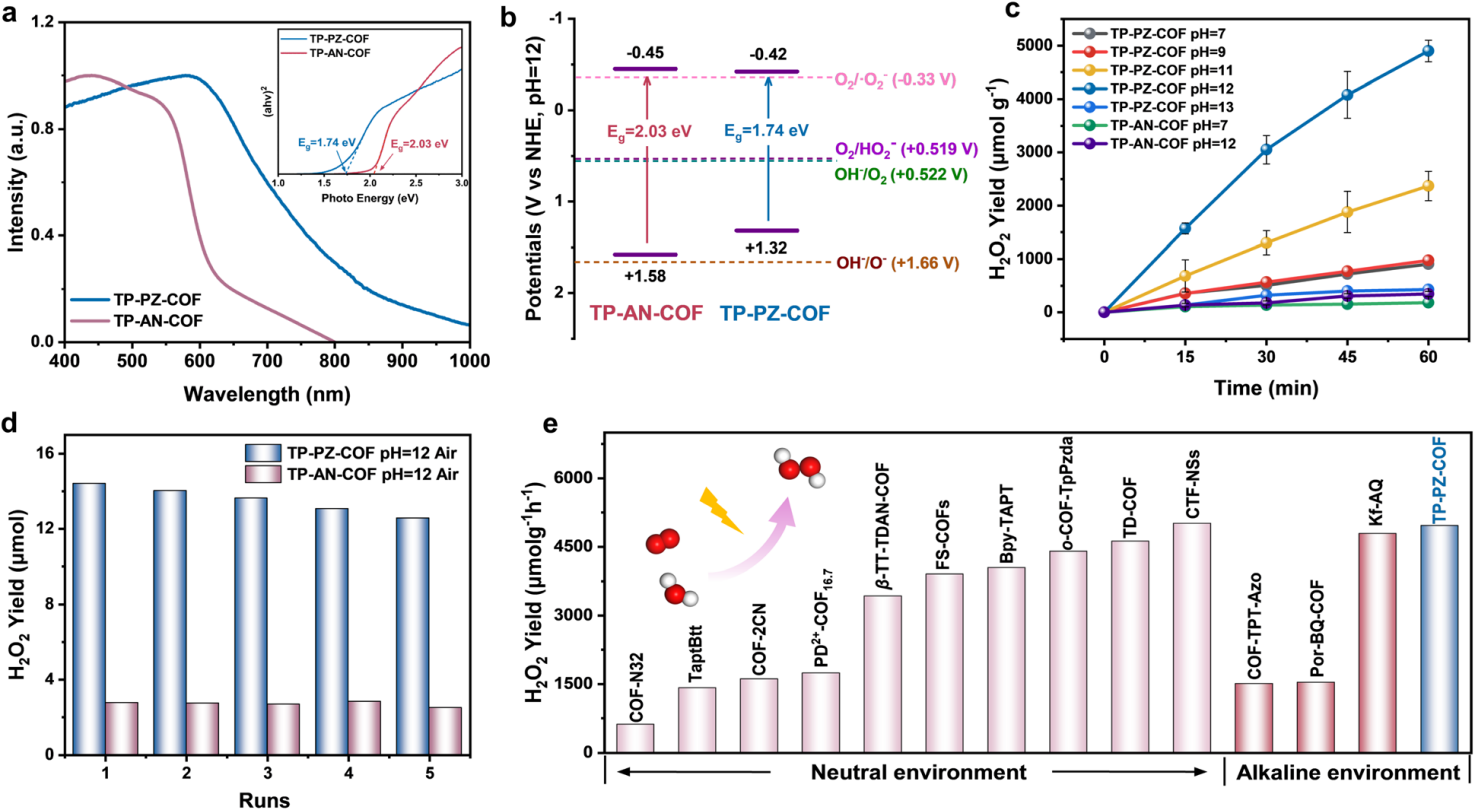
(a) UV/vis diffuse absorption spectra and Tauc plot (inset). (b) Experimentally derived energy band alignments. (c) Photocatalytic H_2_O_2_ yield rates for TP-PZ-COF and TP-AN-COF. (d) Cycling performance of TP-PZ-COF and TP-AN-COF. (e) Summarized H_2_O_2_ production rate in different environments.

The band structure of a photocatalyst, especially the positions of the valence band (VB) and conduction band (CB), critically influences its photocatalytic performance by determining the thermodynamic driving force of the reaction.^36,37^ Since the band-edge positions are pH-dependent, it is essential to characterize both COF materials under their actual operating pH conditions to accurately evaluate their electronic structures.^38^ As shown in Fig. 3a, TP-PZ-COF exhibited a broader UV-vis absorption profile compared to the conventional anthracene-based TP-AN-COF, which could be attributed to the strong electron-withdrawing effect of the nitrogen-containing aromatic heterocycles. Based on the absorption edges, the optical bandgaps of TP-PZ-COF and TP-AN-COF were estimated to be 1.74 and 2.03 eV, respectively (inset in Fig. 3a). The Mott–Schottky plots of both COFs show positive slopes, confirming their n-type semiconducting nature (Fig. S9). Under alkaline conditions (pH = 12) using Ag/AgCl as the reference electrode, the flat-band potentials (*E*_fb_) of TP-PZ-COF and TP-AN-COF were determined to be −0.62 eV and −0.65 eV, respectively. For n-type semiconductors, the CB edge is typically located approximately 0.20 V more negative than the flat-band potential. Accordingly, the CB positions of TP-PZ-COF and TP-AN-COF were estimated to be −0.42 eV and −0.45 eV (*vs.* NHE, pH = 12), respectively. Combining these values with the optical bandgaps, the VB positions were calculated using *E*_VB_ = *E*_CB_ + *E*_g_, yielding 1.32 and 1.58 eV for TP-PZ-COF and TP-AN-COF (*vs.* NHE, pH = 12). Considering the standard redox potentials under alkaline conditions (pH = 12), including O_2_/˙O_2_^−^ (−0.33 eV), O_2_/HO_2_^−^ (+0.519 eV), and OH^−^/O_2_ (−0.522 eV), the band structures of both COFs are thermodynamically favorable for overall photocatalytic H_2_O_2_ production (Fig. 3b).^39,40^ Both materials can facilitate H_2_O_2_ generation through the 2e^−^ ORR (O_2_ + 2e^−^ + H_2_O → HO_2_^−^ + OH^−^) coupled with the 4e^−^ WOR (4OH^−^ → O_2_ + 2H_2_O + 4e^−^).^41^ The photocatalytic H_2_O_2_ production performance of TP-PZ-COF and TP-AN-COF was evaluated under visible-light irradiation (*λ* > 420 nm, 300 W Xe lamp) without any sacrificial agent, in air and NaOH solutions of different pH values (Fig. S1 and S10). The results showed that both COFs exhibited the highest activity at pH = 12. Notably, under O_2_-saturated alkaline conditions (pH = 12), TP-PZ-COF achieved a H_2_O_2_ production rate of 4961 µmol g^−1^ h^−1^ within 1 h, representing an 8.1-fold enhancement compared to TP-AN-COF (606 µmol g^−1^ h^−1^) (Fig. 3c). After normalization by surface area, the H_2_O_2_ production rate of the TP-PZ-COF catalyst reaches 4.8 µmol m^−2^ h^−1^, whereas that of the TP-AN-COF catalyst is only 0.63 µmol m^−2^ h^−1^. Considering that H_2_O_2_ may undergo slight disproportionation under alkaline conditions, we performed control experiments to evaluate its stability at pH 12 (Fig. S11 and S12). In the absence of the catalysts, H_2_O_2_ remained relatively stable in the dark but showed a modest decrease under light irradiation. Upon introducing the COF catalysts, H_2_O_2_ decayed more rapidly; notably, TP-AN-COF induced a more pronounced decline, whereas TP-PZ-COF resulted in a comparatively slower decrease. These results indicated that H_2_O_2_ experiences minor decomposition under alkaline light-irradiated conditions; nevertheless, TP-PZ-COF still delivered higher net H_2_O_2_ accumulation during photocatalysis. Impressively, TP-PZ-COF demonstrated excellent recyclability, retaining high activity over five consecutive photocatalytic cycles with only a slight decline in H_2_O_2_ production (Fig. 3d).

The FT-IR, ^13^C NMR and PXRD spectra displayed no noticeable changes after multiple photocatalytic cycles, further confirming the structural stability of TP-PZ-COF and TP-AN-COF during continuous H_2_O_2_ production in 0.01 M NaOH (Fig. S13–S18). Moreover, its apparent quantum yield (AQY) reached 2.53% at 600 nm, consistent with its visible-light absorption profile (Fig. S19). Under simulated solar illumination (AM 1.5G), TP-PZ-COF achieved a solar-to-chemical conversion (SCC) efficiency of 0.72%, outperforming most previously reported COF-based photocatalysts (Fig. 3e and Table S3). Moreover, TP-PZ-COF exhibited stable H_2_O_2_ production under continuous light irradiation (Fig. S20). Under natural sunlight irradiation (10:30 a.m. to 4:30 p.m.), the system achieved a cumulative H_2_O_2_ yield of 16.6 µmol over 6 h, representing a ∼9.3-fold increase compared to TP-AN-COF (Fig. S21). To enhance practical applicability, TP-PZ-COF was uniformly incorporated into a polyacrylamide (PAAm) hydrogel matrix, affording a COF-hydrogel film with a side length of 2 cm (Fig. S22).^42^ Under simulated sunlight, the resulting film achieved an outstanding H_2_O_2_ production rate of 55.4 mmol h^−1^ m^−2^. Also, the solution collected after 6 hours of light irradiation of the film was applied to simulated industrial dye wastewater containing rhodamine B (Rh B) and rose bengal (RB), exhibiting remarkable purification performance (Fig. S23 and S24).^43^ This strategy may pave the way for the practical application of photocatalytic systems in alkaline aqueous environments. Additionally, we prepared two phenazine-based COFs with different β-ketoenamine densities, DP-PZ-COF and HP-PZ-COF, to probe the influence of β-ketoenamine tautomerization on photocatalytic H_2_O_2_ production under alkaline conditions (Fig. S25 and S26). The H_2_O_2_ generation rates in alkaline media reveal that decreasing the β-ketoenamine density leads to a gradual decline in photocatalytic efficiency. Remarkably, DP-PZ-COF (610 µmol g^−1^ h^−1^) and HP-PZ-COF (456 µmol g^−1^ h^−1^) still outperform TP-AN-COF (340 µmol g^−1^ h^−1^), further highlighting the crucial role of the phenazine unit in modulating molecular orbitals and stabilizing the hydrogen-bond network (Fig. S27).

**Fig. 4:**
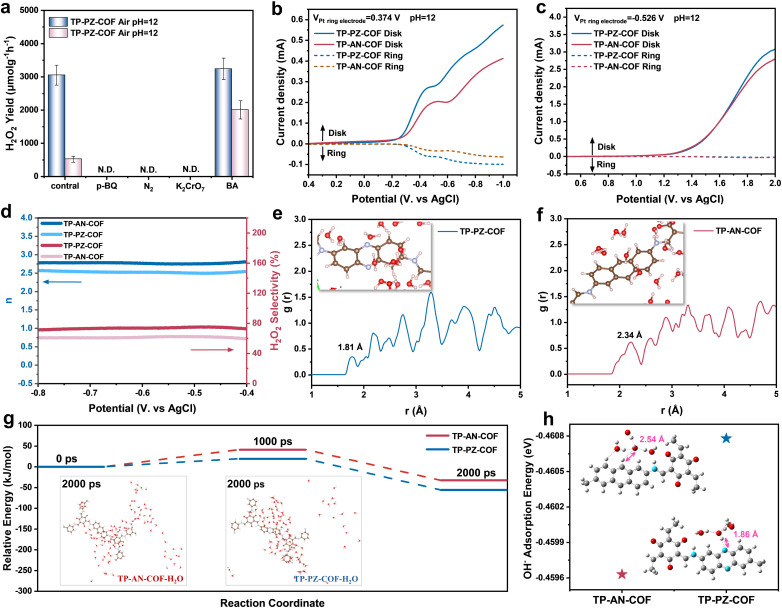
(a) Comparison of H_2_O_2_ production rates by TP-PZ-COF and TP-AN-COF under different conditions. (b) The potential of the Pt ring electrode was set to 0.374 V *vs.* Ag/AgCl for the detection of H_2_O_2_. (c) The potential of the Pt ring electrode was set to −0.526 V *vs.* Ag/AgCl for the detection of O_2_. (d) The ORR electron transfer number calculated from RRDE measurement in O_2_ pre-saturated 0.01 M NaOH and H_2_O_2_ selectivity of TP-AN-COF and TP-PZ-COF. Radial distribution functions (AIMD) of water with respect to the (e) C center of TP-AN-COF and (f) N center of TP-PZ-COF. (g) Time-resolved relative energy profiles of TP-PZ-COF and TP-AN-COF. (h) The adsorption energy of (H_2_O)_3_OH^−^ for TP-PZ-COF and TP-AN-COF.

A series of control experiments were conducted under alkaline conditions to elucidate the reaction pathway of photocatalytic H_2_O_2_ generation.^44^ As shown in Fig. S28, negligible H_2_O_2_ was detected under either an N_2_ atmosphere or dark conditions, confirming that H_2_O_2_ formation in this system was primarily driven by the light-induced 2e^−^ ORR (O_2_ + 2e^−^ + H_2_O → HO_2_^−^ + OH^−^), rather than 2e^−^ WOR (3 OH^−^ → HO_2_^−^ + H_2_O + 2e^−^). To identify the reactive species involved in the redox process, quenching experiments were performed using potassium dichromate (K_2_Cr_2_O_7_), benzyl alcohol (BA), and *p*-benzoquinone (*p*-BQ) as scavengers for electrons (e^−^), holes (h^+^), and superoxide radicals (˙O_2_^−^), respectively (Fig. 4a). In the presence of K_2_Cr_2_O_7_, both COFs produced negligible amounts of H_2_O_2_, corroborating the electron-driven nature of the ORR pathway. Online gas analysis further confirmed that O_2_ evolution *via* the 4e^−^ WOR was remarkably promoted under alkaline conditions with a rate of 18.57 µmol h^−1^, nearly 4-fold higher than that of TP-AN-COF (4.70 µmol h^−1^), which was ascribed to the superior OH^−^ adsorption capacity and lower energy barrier for the 4e^−^ WOR pathway of the PZ unit (Fig. S29). Upon the addition of BA, the H_2_O_2_ production rate of TP-AN-COF increased markedly from 606 to 1957 µmol g^−1^ h^−1^ (3.2-fold), whereas TP-PZ-COF showed only a slight enhancement (from 3003 to 3234 µmol g^−1^ h^−1^), indirectly suggesting that TP-PZ-COF possesses inherently stronger OH^−^ affinity. When excess *p*-BQ was introduced, H_2_O_2_ generation by both COFs was largely suppressed due to the quenching of ˙O_2_^−^, indicating that ˙O_2_^−^ is a key intermediate in the ORR pathway. Electron paramagnetic resonance (EPR) spectroscopy with 5,5-dimethyl-1-pyrroline *N*-oxide (DMPO) as a spin-trapping agent offered independent verification of the generation of ˙O_2_^−^ under light irradiation (Fig. S30), supporting a stepwise 1e^−^ reduction mechanism for H_2_O_2_ formation in both systems. Overall, TP-PZ-COF demonstrated considerably enhanced photocatalytic H_2_O_2_ production efficiency under alkaline conditions compared to TP-AN-COF. Rotating ring-disk electrode (RRDE) analysis revealed that TP-PZ-COF delivered a lower water-generation current and a superior 2e^−^ ORR H_2_O_2_ generation current than TP-AN-COF (Fig. 4b). Moreover, pH-corrected electrochemical analysis of the WOR revealed a significantly higher O_2_ evolution current for TP-PZ-COF compared to TP-AN-COF (Fig. 4c), consistent with its enhanced 4e^−^ WOR activity. According to the Levich equation, the average electron transfer numbers (*n*) during the ORR were calculated to be 2.5 for TP-PZ-COF and 2.8 for TP-AN-COF (Fig. 4d), implying that TP-PZ-COF preferentially undergoes the 2e^−^ ORR pathway. As shown in Fig. 4h, TP-PZ-COF achieved a high H_2_O_2_ selectivity of ∼75% within the potential range of −0.4 to −0.8 V, significantly higher than that of TP-AN-COF (∼62%). This distinct difference highlights the kinetic preference of TP-PZ-COF for the selective 2e^−^ ORR (O_2_ + 2e^−^ + H_2_O → HO_2_^−^ + OH^−^) and 4e^−^ WOR (4OH^−^ → O_2_ + 2H_2_O + 4e^−^) processes, thus enabling more efficient and selective H_2_O_2_ production.

To gain deeper insights into the interactions between (H_2_O)_*n*_OH^−^ clusters, molecular O_2_, and the PZ moieties, AIMD simulations were performed by placing COF fragments in an explicit alkaline aqueous environment.^45,46^ As shown in Fig. 4e and f, radial distribution function (RDF) analysis revealed a significantly shorter hydrogen-bonding distance between H_2_O molecules and the PZ units in TP-PZ-COF (1.81 Å) compared to the AN-based TP-AN-COF (2.34 Å), suggesting stronger hydrogen-bonding interactions. These enhanced interactions likely promoted OH^−^ transport *via* the Grotthuss mechanism, thereby facilitating more efficient photocatalytic H_2_O_2_ production.^47^ Contact angle measurements also confirmed the conclusion regarding the improved surface hydrophilicity of TP-PZ-COF (Fig. S31). Moreover, the relative energies of the two COFs were tracked over a 5.5 ns AIMD simulation (Fig. 4g, S32 and S33). At 2000 ps, TP-PZ-COF exhibited a lower energy state and a higher number of surrounding water molecules, suggesting stronger interactions with the adjacent (H_2_O)_*n*_OH^−^ clusters. Consistent with these findings, DFT calculations demonstrated that TP-PZ-COF showed a superior binding affinity toward the (H_2_O)_3_OH^−^ cluster at its PZ sites (Fig. 4i). These results align well with the superior 4e^−^ WOR performance of TP-PZ-COF and underscore the critical role of PZ moieties in enhancing OH^−^ adsorption and transport under alkaline conditions.^48^

**Fig. 5:**
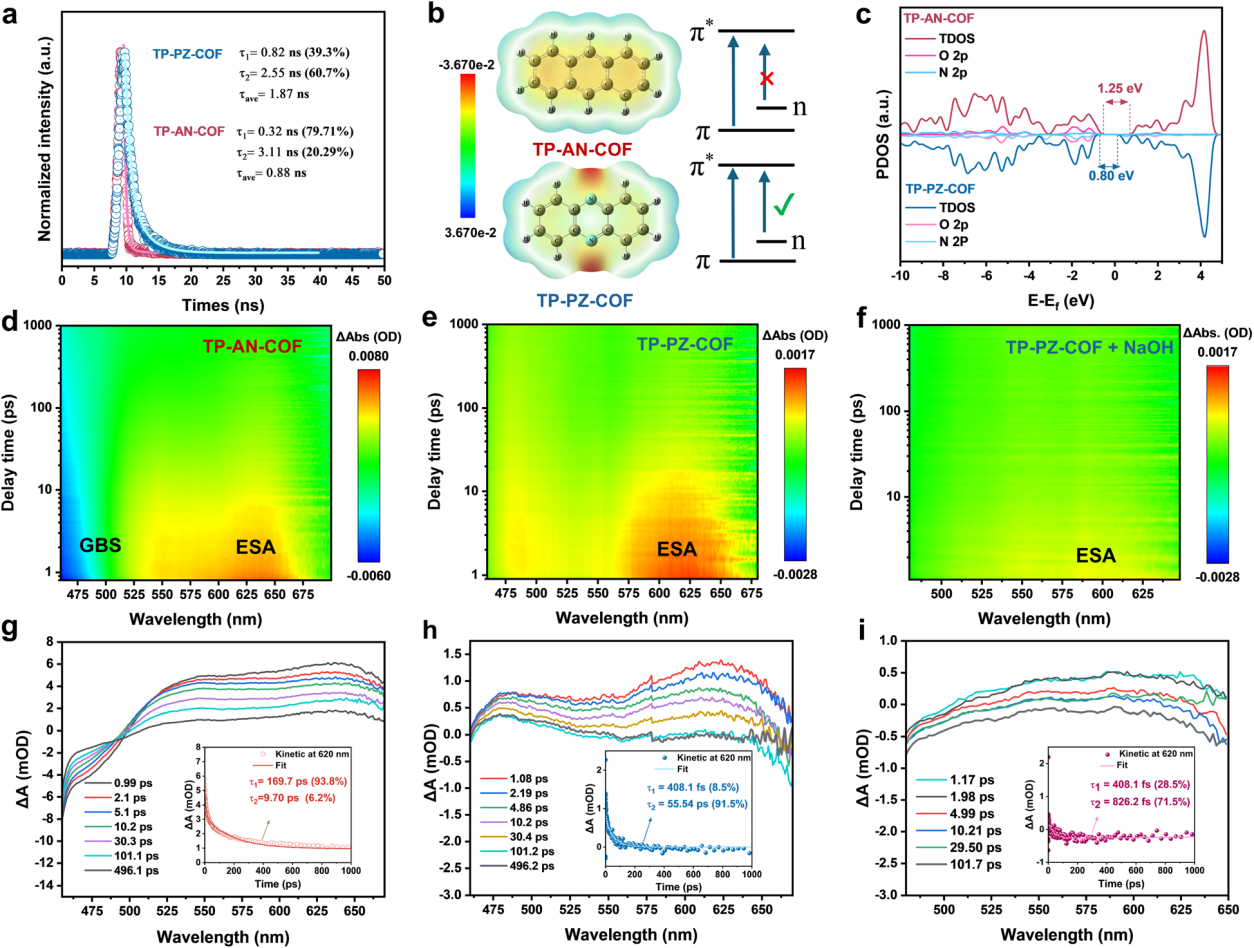
(a) Time-resolved PL decay spectra of TP-PZ-COF and TP-AN-COF, excited at 375 nm. (b) Electrostatic surface potential maps and molecular orbital transitions of the PZ unit and anthracene unit. (c) PDOS analysis for TP-PZ-COF and TP-AN-COF. fs-TA two-dimensional pseudocolor maps of (d) TP-AN-COF, (e) TP-PZ-COF, and (f) TP-PZ-COF dispersed in 0.01 M NaOH aqueous solution. Kinetic traces at 620 nm extracted from the femtosecond transient absorption spectra of (g) TP-AN-COF, (h) TP-PZ-COF, and (i) TP-PZ-COF in 0.01 M NaOH.

The origin of the distinct photocatalytic activities of the two COFs was further elucidated by systematically probing their charge-carrier dynamics under alkaline conditions. Electrochemical impedance spectroscopy (EIS) and transient photocurrent (*i*–*t*) measurements show that TP-PZ-COF delivers a markedly higher photocurrent response and a lower charge-transfer resistance than TP-AN-COF, consistent with more efficient charge transport and interfacial charge transfer (Fig. S34 and S35). In addition, photoluminescence (PL) spectroscopy revealed a substantially weaker emission for TP-PZ-COF (Fig. S36), suggesting suppressed radiative recombination and enhanced charge separation. These observations are further supported by fluorescence lifetime (FLT) measurements, where TP-PZ-COF exhibited a longer excited-state lifetime than TP-AN-COF, indicative of a higher fraction of long-lived charge carriers (Fig. 5a). DFT calculations provided additional insights into the electronic structures. In addition to the typical π → π* transitions of aromatic rings, the sp^2^-hybridized nitrogen atoms in the PZ units of TP-PZ-COF possess non-bonding lone-pair electrons, enabling n → π* transitions with adjacent aromatic carbons.^49^ This orbital coupling lowers the excitation energy and is expected to promote preferential localization of photoexcited electrons on the PZ moieties (Fig. 5b). The longer *τ*_2_ indicates a slower carrier recombination process, while the partial density of states (PDOS) analysis revealed an increased density of p-orbital electrons near the valence band edge in TP-PZ-COF, effectively narrowing the bandgap and facilitating exciton dissociation (Fig. 5c).

To investigate the impact of the n → π* transition on excited-state carrier dynamics in TP-PZ-COF and TP-AN-COF, fs-TAS was employed. Upon excitation with a 420 nm pump pulse, two-dimensional pseudocolor maps (Fig. 5d and e) and representative decay profiles over a delay range of 1–500 ps (Fig. 5g and h) were obtained. For TP-AN-COF, a distinct ground-state bleaching (GSB) signal was observed at 475 nm, along with an excited-state absorption (ESA) band at 620 nm. In contrast, TP-PZ-COF exhibited positive ESA signals at both 475 and 620 nm. Kinetic fitting of the time-resolved signals at 620 nm using a biexponential model yielded two decay components for each sample. For TP-AN-COF, the lifetimes were *τ*_1_ = 169.7 ps (93.8%) and *τ*_2_ = 9.70 ps (6.2%) (Fig. 5g, inset), while TP-PZ-COF showed *τ*_1_ = 408.1 fs (8.5%) and *τ*_2_ = 55.54 ps (91.5%) (Fig. 5h, inset). The shorter *τ*_1_ for TP-PZ-COF may be associated with faster relaxation of hot electrons toward the CB minimum, thereby potentially facilitating the availability of photogenerated electrons for subsequent redox processes. In comparison, TP-PZ-COF shows a noticeably longer *τ*_2_ than TP-AN-COF, suggesting an increased population of longer-lived charge carriers that could reach the surface and contribute to the ORR and WOR. Moreover, upon addition of 10% NaOH solution (pH = 12), the ESA signal of TP-PZ-COF was significantly attenuated (Fig. 5i), accompanied by a sharp decrease in *τ*_2_ to 826.2 fs (71.5%), indicating efficient quenching of photoexcited electrons by OH^−^ species. Interestingly, visible O_2_ bubble formation was observed under 420 nm laser irradiation, confirming the photocatalytic activity (Fig. S37). Collectively, these findings highlight the superior charge separation efficiency and extended lifetime of active electrons in TP-PZ-COF, contributing to enhanced photocatalytic performance.

**Fig. 6:**
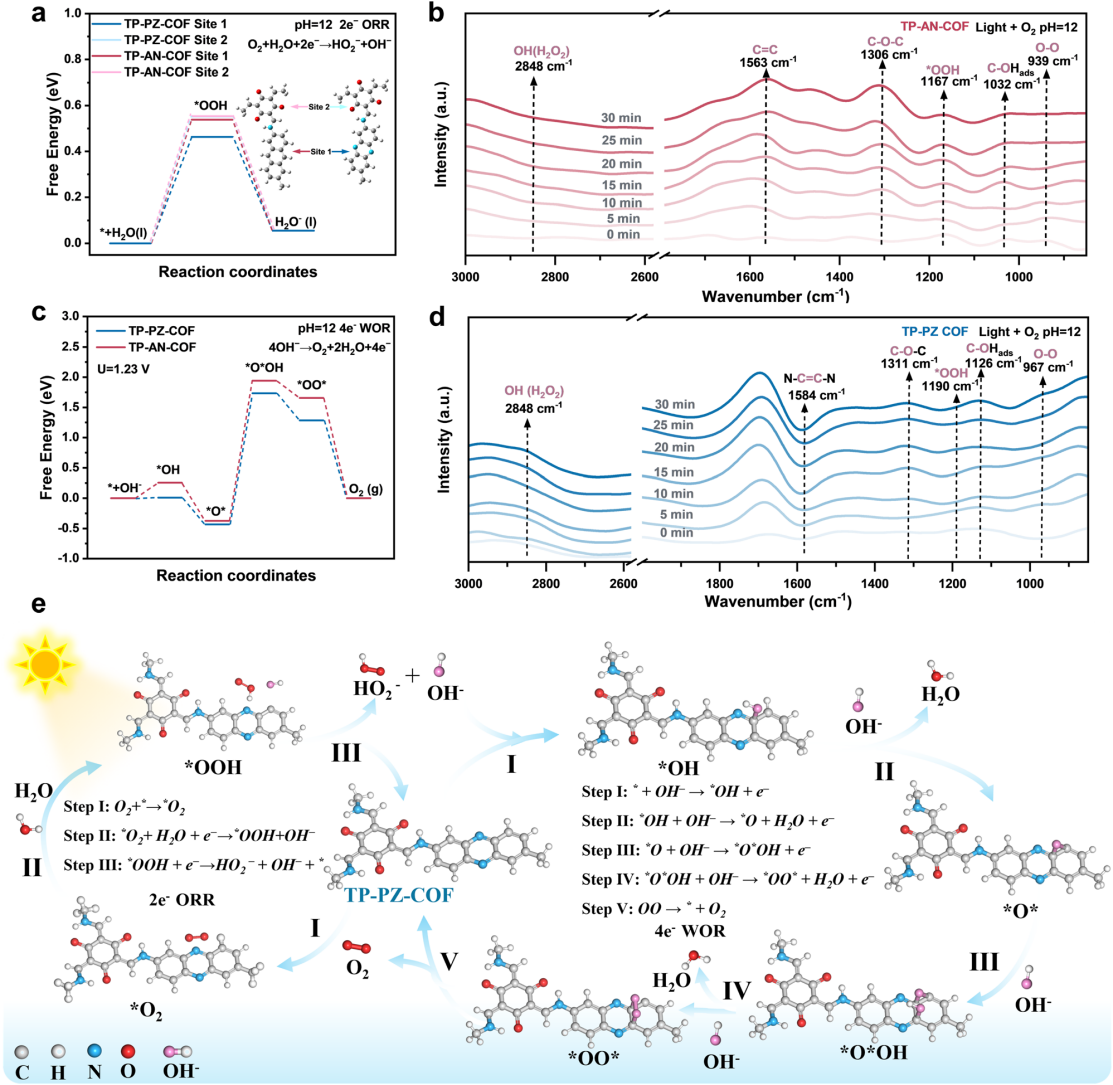
(a) Free-energy diagrams for a stepwise 1e^−^ ORR to H_2_O_2_ on TP-AN-COF and TP-PZ-COF. (b) *In situ* DRIFT spectra recorded during photocatalytic H_2_O_2_ production on TP-AN-COF. (c) Calculated free energy diagrams of the WOR pathway in both COFs. (d) *In situ* DRIFT spectra recorded during photocatalytic H_2_O_2_ production on TP-AN-COF. (e) Schematic illustration of the overall H_2_O_2_ production mechanism over TP-PZ-COF.

Guided by DFT calculations and spectroscopic analyses, the critical role of PZ units in modulating electron distribution and charge transfer in TP-PZ-COF was established, prompting investigations to elucidate its catalytic mechanism for H_2_O_2_ production under alkaline conditions. Experimental results demonstrated that both COFs followed a stepwise 1e^−^ ORR pathway involving molecular oxygen and intermediates such as *OOH (O_2_ + H_2_O +1e^−^ → *OOH + OH^−^; *OOH + 1e^−^ → HO_2_^−^). To probe the intrinsic differences in ORR active sites between the two materials, detailed Gibbs free energy (Δ*G*) analyses were performed. In TP-AN-COF, the TFP moiety revealed a more favorable Δ*G* for *OOH formation compared to the AN unit, suggesting it as the predominant ORR active site (Fig. 6a).^50^ In contrast, the PZ moiety in TP-PZ-COF was identified as the most favorable site, highlighting its superior capability for H_2_O and O_2_ adsorption and activation under alkaline conditions. These results were further corroborated by FTIR spectroscopy. Upon immersion in water, the characteristic phenazine-associated peak in TP-PZ-COF exhibited a redshift from 1163.5 cm^−1^ to 1069.4 cm^−1^, whereas the peaks corresponding to AN units remained unchanged (Fig. S38 and S39). This spectral shift indicates strong N⋯H–O hydrogen bonding interactions at the PZ site, facilitating *H dissociation and subsequent O_2_ activation to form *OOH intermediates. Furthermore, DRIFTS measurements provided direct evidence for the stepwise 1e^−^ ORR mechanism. As shown in Fig. 6b and d, characteristic *OOH signals appeared at ∼1167 and ∼1190 cm^−1^, while O–O vibrational modes were observed at ∼939 and ∼967 cm^−1^ in TP-AN-COF and TP-PZ-COF, respectively.^51^ Also, a newly emerging peak at ∼2838 cm^−1^, attributed to the O–H stretching vibration of H_2_O_2_, increased in intensity under visible light irradiation over time, serving as direct spectroscopic evidence for photocatalytic H_2_O_2_ production.^52^

Under alkaline conditions, the 4e^−^ WOR leading to O_2_ evolution becomes thermodynamically more favorable compared to neutral environments.^53^ In contrast to neutral conditions that generally necessitate water deprotonation, the alkaline WOR pathway proceeds *via* direct OH^−^ adsorption, yielding *OH intermediates, which subsequently evolve through *O*, *O*OH, and *OO* species in a stepwise manner.^54^ DFT calculations revealed that, in TP-PZ-COF, the C atoms adjacent to N atoms within the PZ units possess more positive electrostatic potential due to the high electronegativity of N (Fig. S40). As a result, the *OH formation on the PZ sites of TP-PZ-COF is more favorable than on the conventional AN moiety, with a reduced Δ*G* by approximately 0.25 eV. This finding suggests that the incorporation of PZ units effectively modulates the WOR energy landscape, thereby enhancing the overall H_2_O_2_ production efficiency. Additional evidence was provided by *in situ* DRIFTS measurements. As shown in Fig. 6b and d, the absorption bands at ∼1032 and ∼1126 cm^−1^ are attributed to surface-bound C–OH_ads_ intermediates, confirming the initial OH^−^ adsorption step in the WOR process. In addition, a distinct vibration at ∼1300 cm^−1^ corresponds to the formation of C–O–C species, indicating the generation of *O* intermediates in both COFs.^55^ Notably, the pronounced attenuation of the C=C stretching vibration at ∼1584 cm^−1^, attributed to the PZ moiety, upon photoirradiation further substantiates the involvement of aromatic C=C as active sites in the 4e^−^ WOR pathway for O_2_ evolution (4OH^−^ → O_2_ + 2H_2_O + 4e^−^).

Combined insights from *in situ* spectroscopic analyses and DFT calculations provide an improved understanding of the H_2_O_2_ formation pathways in both COFs. As illustrated in Fig. S41 and 6e, a mechanistic model is proposed for the photocatalytic production of H_2_O_2_ over TP-AN-COF and TP-PZ-COF under alkaline conditions. In TP-AN-COF, the hydrophilic TFP unit is identified as the active site for a stepwise 1e^−^ ORR pathway, where surface C=O moieties initially form weak hydrogen bonds with H_2_O, facilitating *H capture by O_2_ to generate the *OOH intermediate, which then dissociates into HO_2_^−^ and OH^−^ (O_2_ + H_2_O +1e^−^ → *OOH + OH^−^; *OOH + 1e^−^ → HO_2_^−^). The liberated OH^−^ species can be adsorbed by the aromatic rings on the AN unit, promoting a 4e^−^ WOR to evolve O_2_ (4OH^−^ → O_2_ + 2H_2_O + 4e^−^). Notably, the evolved O_2_ may be reused in the ORR, forming a catalytic cycle. In contrast, the PZ unit in TP-PZ-COF is proposed to act as a bifunctional active site that simultaneously promotes the 2e^−^ ORR and the 4e^−^ WOR. The nitrogen atoms in the PZ ring can stabilize O_2_ and (H_2_O)_*n*_OH^−^*via* strong hydrogen bonding, which promotes the formation of *OOH intermediates and their subsequent dissociation into HO_2_^−^ and OH^−^. The resulting OH^−^ is favorably adsorbed by adjacent carbon atoms, forming *OH intermediates that proceed through *O*OH and *OO* species to produce O_2_. This O_2_ can subsequently re-enter the ORR cycle. Such cooperative ORR/WOR catalytic behavior centered on the PZ moiety is key to the superior H_2_O_2_ production efficiency observed for TP-PZ-COF under alkaline conditions. These findings underscore the pivotal role of the PZ unit in H_2_O_2_-oriented COF design, offering a dual advantage of enhanced charge carrier kinetics and robust hydrogen-bonding frameworks.

## Conclusion

By employing a dual-regulation strategy of molecular orbital engineering and hydrogen-bonding network design, we successfully designed and synthesized a phenazine-based covalent organic framework (TP-PZ-COF) capable of efficient overall photocatalytic H_2_O_2_ production from alkaline aqueous solution and O_2_*via* a synergistic 2e^−^ ORR and 4e^−^ WOR. Experimental results revealed that the PZ moiety plays a crucial role in enhancing photogenerated charge utilization and constructing a stable hydrogen-bonding network, thereby facilitating efficient exciton separation and directional OH^−^ transport, which collectively boost photocatalytic H_2_O_2_ production. Further DFT and AIMD calculations confirmed that the incorporation of the PZ unit significantly lowers the energy barriers for both the stepwise 1e^−^ ORR pathway to form H_2_O_2_ and the 4e^−^ WOR pathway to evolve O_2_, providing a theoretical basis for the excellent photocatalytic activity of TP-PZ-COF. Moreover, the integration of TP-PZ-COF with polyacrylamide further expands its application potential in the purification of industrial dye wastewater. This work not only offers a new perspective for sustainable H_2_O_2_ production in basic media but also provides valuable insights into the rational design of metal-free COF-based photocatalysts for O_2_-to-H_2_O_2_ conversion.

## Author contributions

Z. Yu conceived and designed the research. J. Zhang and X. Zhang assisted in catalyst synthesis and conducted the photocatalytic activity tests. X. Sun and Z. Yu carried out the material characterization and fs-TAS measurements. G. W. performed the DFT calculations. Z. Yu wrote the manuscript under the supervision of F. Yu and J. Hua. All authors discussed the results and contributed to the manuscript revision.

## Conflicts of interest

The authors declare no conflicts of interest.

## Supplementary Material

SC-OLF-D5SC08298F-s001

## Data Availability

The data that support the findings of this study are available from the corresponding author upon reasonable request. Supplementary information (SI) is available. See DOI: https://doi.org/10.1039/d5sc08298f.
