# Supplementary material for: Alkaline-adaptive covalent organic framework photocatalysts: synergistic molecular orbital and hydrogen-bond network engineering for H2O2 production
Source: Chem Sci. 2026 Jan 29;17(13):6456–66. doi: 10.1039/d5sc08298f (PMC12875404; doi:10.1039/d5sc08298f)
Supplement: SC-017-D5SC08298F-s001 [file SC-017-D5SC08298F-s001.pdf]

Supplementary Information (SI) for Chemical Science.
This journal is © The Royal Society of Chemistry 2026

Supporting information

Alkaline-adaptive covalent organic framework photocatalysts: synergistic molecular orbital and hydrogen-bond network engineering for H_2O_2 production

Zhiwu Yu, [a] *Jiayi Zhang,* [a] *Xiaolong Zhang,* [a] *Xuwen Sun,* [a] *Zhiyun Zhang,* [a] *Guihong Wu,* [b] *Fengtao Yu*[*b] *and Jianli Hua*[*a]

[a] Key Laboratory for Advanced Materials and Joint International Research Laboratory for Precision Chemistry and Molecular Engineering, Feringa Nobel Prize Scientist Joint Research Center, Frontiers Science Center for Materiobiology and Dynamic Chemistry, School of Chemistry and Molecular Engineering, East China University of Science and Technology Shanghai, 200237, People's Republic of China

E-mail: jlhua@ecust.edu.cn

[b] Jiangxi Province Key Laboratory of Functional Organic Polymers, East China University of Technology, Nanchang 330013, People's Republic of China.

E-mail: fty853815622@ecut.edu.cn

Table of Contents

Section S1. Materials and Methods 4
S1.1 Materials 4
S1.2 Instruments 4
S1.3 Photocatalytic H_2O_2 measurements 5
S1.4 Photocatalytic Decomposition of H_2O_2 over COFs under Alkaline Conditions 5
S1.5 Quenching experiments measurements 5
S1.6 Photocatalytic Water Oxidation Reaction (WOR) Test 6
S1.7 Apparent quantum yield (AQY) measurement 6
S1.8 SCC efficiency measurements 6
S1.9 Electrochemical rotating ring-disk electrode (RRDE) measurement 7
S1.10 In-situ diffuse reflectance infrared fourier transform spectroscopy (DRIFTS) measurements 7
Fig. S2 8
S1.11 Degradation of Rh B and RB in dye wastewater containing metal ions 8
S1.12 Computational Methods 9
S1.13 Gibbs Free Energy Calculations 9
S1.14 Synthesis of 2,7-diaminophenazine·benzylideneaniline (DAPH·Bnzph) 10
S1.15 Synthesis of TP-PZ COF 11
S1.16 Synthesis of Tp-AN-COF 11
Section S2. Characterization Fig.s 13
Fig. S3 13
Fig. S4 13
Fig. S5 14
Fig. S6 14
Fig. S7 15
Fig. S8 15
Fig. S9 16
Fig. S10 16
Fig. S11 17
Fig. S12 17
Fig. S13 18
Fig. S14 18
Fig. S15 19
Fig. S16 19

Fig. S17 20
Fig. S18 20
Fig. S19 21
Fig. S20 22
Fig. S21. 22
Fig. S22 23
Fig. S23 24
Fig. S24 24
Fig. S25 25
Fig. S26 26
Fig. S27 27
Fig. S28 27
Fig. S29 28
Fig. S30 28
Fig. S31 29
Fig. S32 30
Fig. S33 30
Fig. S34 31
Fig. S35 31
Fig. S36 32
Fig. S37 32
Fig. S38 33
Fig. S39 33
Fig. S40 34
Fig. S41 35
Section S3. Supplementary Tables 36
Table S1. Fractional atomic coordinates and the unit cell of TP-AN-COF 36
Table S2. Fractional atomic coordinates and the unit cell of TP-PZ-COF 37
Table S3. Comparison of photocatalytic performances among recently reported COFs 38
Section S4. Supporting References 40

Section S1. Materials and Methods

S1.1 Materials

2,7-Dibromophenazine was purchased from Shanghai Haohong Biomedical Technology Co., Ltd. Tris(dibenzylideneacetone)dipalladium(0) was obtained from Shanghai Morn Chemical Technology Co., Ltd. Sodium tert-butoxide was sourced from Beijing Innochem Technology Co., Ltd. Benzophenone imine was purchased from Shanghai Aladdin Biochemical Technology Co., Ltd., and (±)-BINAP was supplied by Beijing Hwailichem Co., Ltd. 1,3,5-Triformylphloroglucinol, 2,4-dihydroxy-1,3,5-benzenetricarboxaldehyde, and 2-hydroxy-1,3,5-benzenetricarboxaldehyde were obtained from Shanghai Tensus Biotechnology Co., Ltd. Additionally, all solvents and reagents, including ethanol, tetrahydrofuran (THF), acetone, methanol (MeOH), benzyl alcohol, 1,4-dioxane, potassium dichromate ($K_2Cr_2O_7$), sodium hydroxide (NaOH), Dimethyl sulfoxide(DMSO) and trimethylbenzene, were purchased from Aladdin Reagent Co., Ltd. All chemicals had a purity of at least 95% and were used as received without further purification.

S1.2 Instruments

PXRD data were collected on a Bruker D8 Advance Powder X-ray Diffractometer using a Cu Kα source (λ = 1.5418 Å) over the range of $2\theta = 2.0–30.0°$ with a step size of 0.02° and 2 s per step. Solid-state ^{13}C-NMR spectra were acquired with the Bruker Advance III 500 MHz spectrometer. Fourier transform infrared (FT-IR) spectra were recorded using KBr pellets on a Nicolet Impact 410 spectrometer. The sample morphology was examined using both a field emission scanning electron microscope (FESEM, JSM-6360LV) and a transmission electron microscope (TEM, JEOL JEM-2100). X-ray photoelectron spectroscopy (XPS) analysis was performed with an ESCALAB 250Xi Thermo Scientific TM XPS equipment. Nitrogen adsorption and desorption at 77 K were carried out using a Micromeritics ASAP 2020 instrument, with the samples being degassed at 120 °C for 12 hours under a vacuum of 10^{-5} bar prior to analysis. The specific surface areas were calculated using the Brunauer-Emmett-Teller (BET) method, and the pore size distribution was determined from the sorption curve using the non-local density functional theory (NLDFT) model. Diffuse reflectance spectroscopy (DRS) measurements were performed with a Varian Cary 500 spectrophotometer. Electron spin resonance (ESR) spectra were recorded using the Bruker EMX nano instrument. Photoluminescence (PL) spectra were collected using a Hitachi F-4500 fluorescence spectrophotometer. All electrochemical tests were conducted in a standard three-electrode cell using a CHI760E S3S4 electrochemical workstation.

S1.3 Photocatalytic H_2O_2 measurements

5.0 mg photocatalyst was dispersed in 50 mL of solution. 3 M NaOH solution was added to adjust the pH to 12. Subsequently, the dispersion underwent ultrasonic treatment for 10 minutes, followed by continuous stirring for 30 minutes. All photocatalytic reactions were conducted in an air atmosphere or O_2-saturated environment under the illumination of a 300 W Xenon Lamp, with a UV cut-off filter at 420 nm. At 15-minute intervals, 3 mL of the solution was sampled. To this solution, 1 mL of 0.1 molL^{-1} aqueous potassium hydrogen phthalate ($C_8H_5KO_4$) solution, 0.1mL 3M HNO_3 and 1 mL of 0.4 molL^{-1} aqueous potassium iodide (KI) solution was added, and the mixture was allowed to sit for 3 hours. Under acidic conditions ($H_2O_2 + 3I^- + 2H^+ \rightarrow I_3^- + 2H_2O$), H_2O_2 molecules reacted with iodide anions (I^-) to produce triiodide anions (I_3^-) with strong absorption around 350 nm. The quantity of I_3^- was determined via UV–vis spectroscopy based on the absorbance at 350 nm, which allowed the estimation of the amount of H_2O_2 produced during each reaction (see below Fig. a and b).[1]

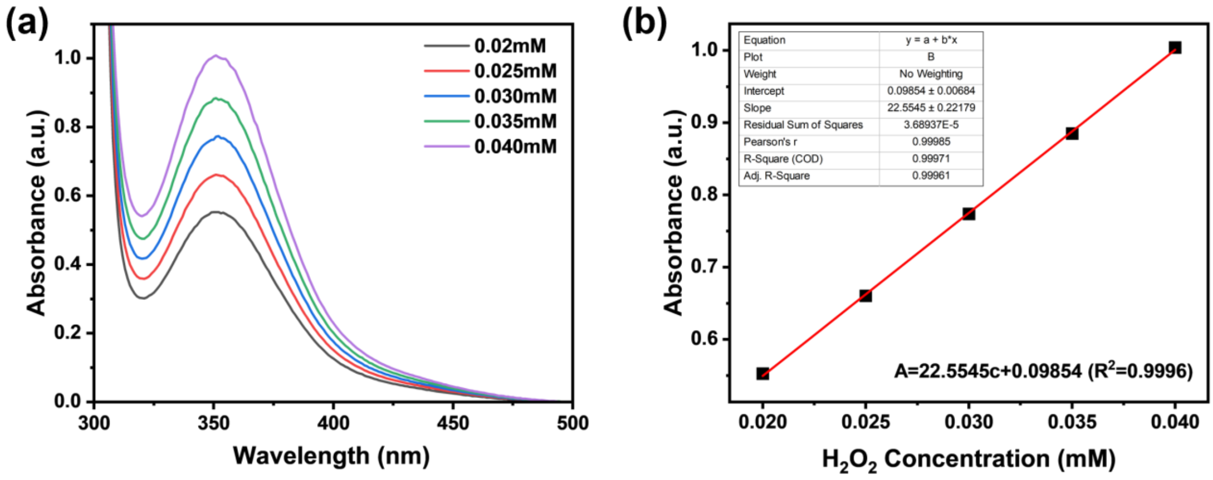

Fig. S1 (a, b) the standard curve used for determining H_2O_2 concentration via the iodometric method.

S1.4 Photocatalytic Decomposition of H_2O_2 over COFs under Alkaline Conditions

Under a nitrogen atmosphere, 5.0 mg of the catalyst was dispersed in 50 mL of 0.01 M NaOH solution containing 1 mM H_2O_2 for photocatalytic decomposition. A 300 W xenon lamp (Zhongjiao Jinyuan, Beijing) was used as the light source, and a cutoff filter was applied to provide visible-light irradiation ($\lambda > 420$ nm, average intensity: 100 mWcm^{-2}).

S1.5 Quenching experiments measurements

5.0 mg photocatalyst was dispersed in 50 mL of solution. 3 M NaOH solution was added to adjust the pH to 12. Subsequently, 100.0 mg of excess $K_2Cr_2O_7$ was added to the dispersion as an electron sacrificial agent; 100.0 mg

of excess p-benzoquinone was added to the dispersion as a superoxide radical scavenger; 10% BA was used as a hole (h^+) scavenger. The dispersion underwent ultrasonic treatment for 10 minutes, followed by continuous stirring for 30 minutes.

S1.6 Photocatalytic Water Oxidation Reaction (WOR) Test

Photocatalytic water oxidation was performed by dispersing 10.0 mg of photocatalyst in 50 mL of 0.01 M NaOH solution containing 0.01 M $K_2Cr_2O_7$ (as an electron acceptor). The reaction was carried out in a sealed gas-phase monitoring system composed primarily of airtight components. The suspension was ultrasonicated for 30 minutes to ensure thorough dispersion. Prior to irradiation, the system was evacuated using a vacuum pump for 30 minutes to remove residual air. A 300 W xenon lamp (Zhongjiao Jinyuan, Beijing) was used as the light source, and a cutoff filter was applied to achieve visible-light irradiation (λ > 420 nm, average intensity: 100 $mWcm^{-2}$). The evolved O_2 during the reaction was carried by the internal gas stream to the gas chromatograph for real-time detection.[2]

S1.7 Apparent quantum yield (AQY) measurement

The AQY of H_2O_2 over TP-PZ-COF was measured with irradiation light through different wavelength bandpass filter (420, 475, 500, 600 and 700 nm).[3] The photon flux of incident light was measured by a PL.MW200 photoradiometer (Zhongjiao Jinyuan, Beijing). The AQY was calculated according to the following equation:

$$\mathrm{AQY}(\%) = \frac{2 \times \text{Number of evolved } \mathrm{H_2O_2} \text{ molecules}}{\text{Number of incident photons}} \times\ 100\% = \frac{2 \times C \times N_A}{S \times P \times \frac{\lambda}{h \times c}} \times\ 100\%$$

Where C is the H_2O_2 production amount (μmol) per hour; N_A is the Avogadro constant (6.02×10^{23} mol^{-1}); S is the irradiation area (4 cm^2); P is the monochromatic light intensity (W cm^{-2}) (P is detected by optical power meter); t is the light irradiation time (3600 s); λ is the wavelength of the monochromatic light (nm); h is the Plank constant (6.626×10^{-34} J s); c is the speed of light (3×10^8 m s^{-1}).

S1.8 SCC efficiency measurements

The solar-to-chemical energy conversion (SCC) efficiency was determined by using an AM 1.5 G solar simulator as the light source (300 W Xe lamp). The photocatalytic reaction was carried out in pure deionized water (10 mL) with photocatalysts (50.0 mg). Ultrasonication and O_2 bubbling for half an hour respectively. During the photocatalytic tests, O_2 was continuously bubbled into the reaction vessel. The solar-to-chemical conversion (SCC) efficiency was calculated using the following equation:

$$\text{SCC efficiency } (\%) = \frac{[\Delta \mathrm{G} \text{ for} \mathrm{H_2O_2} \text{generation } (\mathrm{J\ mol^{-1}}\ )][\mathrm{H_2O_2} \text{ formed (mol)}]}{[\text{Total input power(W)}][\text{Reaction time (s)}]} \times\ 100\%$$

Where $\Delta G_{H_2O_2}$ = 117 kJ mol^{-1} is the free energy for H_2O_2 generation (117 kJ mol^{-1}). The overall irradiation intensity of the AM 1.5 global spectrum (300–2500 nm) is 2030 W m^{-2} and the irradiation areas are 4 × 10^{-4} m^2.

S1.9 Electrochemical rotating ring-disk electrode (RRDE) measurement

The rotating ring disk electrode (RRDE, electrode area: 0.2475 cm^2) was employed as the substrate for the working electrode. The preparation of the working electrode involves first weighing 10.0 mg of the sample and adding it to a mixture of 0.5 mL ethanol and 20 μL of 5 wt% Nafion solution. The mixture is sonicated for 10 minutes to ensure uniform dispersion. Then, 20 μL of the resulting ink is dropped onto a clean disk electrode, repeated twice, and allowed to air dry naturally before testing. The ORR catalytic test is then conducted using a rotating disk electrode as the substrate for the working electrode. A carbon rod serves as the counter electrode, and an Ag/AgCl electrode is used as the reference electrode. The potential is scanned from -1.0 to 0.6 V (vs. Ag/AgCl). The working electrode is placed in an oxygen-saturated 0.1 M phosphate-buffered saline (PBS, pH = 7) solution and 0.01 M NaOH (pH=12), and linear sweep voltammograms (LSV) is performed at a scan rate of 10 mV/s until the curve stabilizes at 1000 rpm. The transfer number electron (n) was calculated by the disk current (I_d) and ring current (I_r) results as the following equations:

$$n = 4 \times \frac{I_d}{I_d + I_r/N}$$

$$H_2O_2\% = 200 \times \frac{I_r/N}{I_d + I_r/N}$$

Where I_d and I_r are the disk and ring current (mA), respectively, N is the collection efficiency of the Pt ring (N = 0.37).[4]

S1.10 In-situ diffuse reflectance infrared fourier transform spectroscopy (DRIFTS) measurements

In-situ DRIFT measurements were carried out on a Nicolet 560 Fourier-transform infrared (FTIR) spectrometer equipped with a Harrick Scientific in situ diffuse reflectance cell and a liquid-nitrogen-cooled mercury cadmium telluride (MCT) detector. Prior to the measurements, 20 mg of the powdered sample was dried at 150 °C to remove adsorbed moisture and then cooled to room temperature. The dried sample was gently ground with an appropriate amount of dry KBr (approximately two spatulas) to ensure homogeneous dispersion and then loaded into the customized in situ cell. Subsequently, approximately half a drop of deionized water was added to slightly moisten the sample surface, simulating the aqueous reaction environment. A background spectrum was recorded before introducing the reaction gases. Argon was then purged through the in-situ cell for 30 min to eliminate residual air,

and FTIR spectra were collected every 5 min during this process. Subsequently, O_2 was introduced for 30 min, with spectra recorded at 5 min intervals, to establish an O_2-saturated atmosphere. After stopping the gas flow, the in-situ cell was sealed. Photocatalytic measurements were initiated by illuminating the sample with an external white-light source. FTIR spectra were collected every 5 min during a total illumination period of 60 min. After the light was switched off, argon was again purged through the cell for an additional 30 min, and spectra were continuously recorded at 5 min intervals. All spectra were processed and presented as difference spectra relative to the initial background, enabling clear visualization of light-induced surface species and their dynamic evolution. In addition, photographs of the actual *in-situ* DRIFT experimental setup are provided in the figure below to more clearly illustrate the measurement configuration and experimental details.

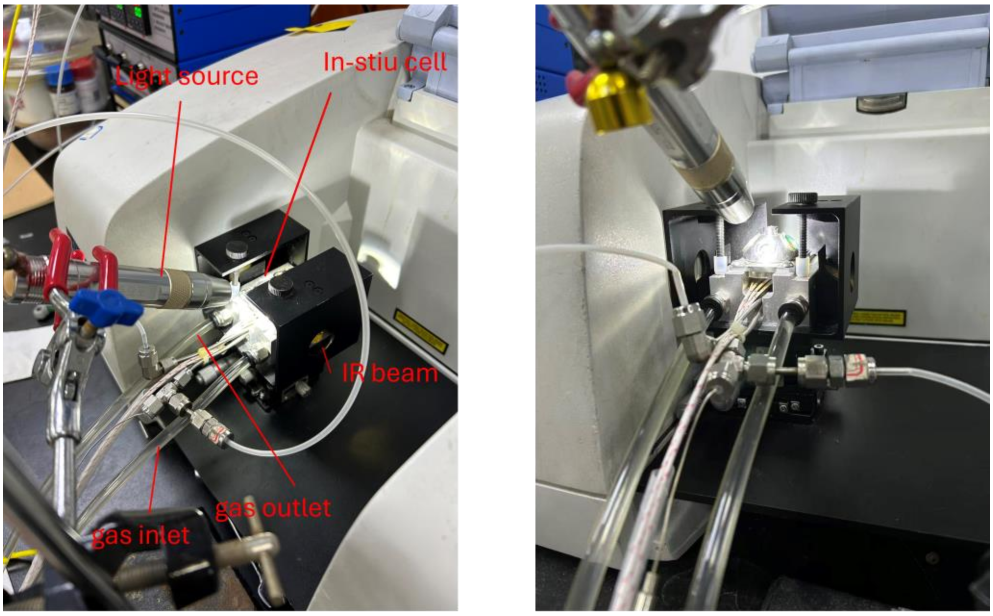

Top-view Front-view

Fig. S2 Top-view and front-view of the *in-situ* DRIFT setup, showing the optical path, gas inlet/outlet, and diffuse reflectance configuration.

S1.11 Degradation of Rh B and RB in dye wastewater containing metal ions

Initially, 10.0 mg L^{-1} solutions of Rhodamine B (Rh B) and Rose Bengal (RB) were separately prepared, each containing 0.1 M $FeSO_4 \cdot 7H_2O$, 0.05 M $CuSO_4$, 0.05 M $Fe_2(SO_4)_3$, or 0.05 M $ZnSO_4$. Subsequently, 1 mL of the prepared Rh B and RB solution was transferred into a 3 mL colorimetric tube, followed by the gradual addition of 2 mL photocatalytically generated H_2O_2 solution—obtained by irradiating 4 cm^2 of TP-PZ-COF hydrogel film in 25 mL of 0.01 M NaOH under a Xe lamp ($\lambda > 420$ nm). The color changes and absorbance of Rh B and RB were then monitored and recorded.

S1.12 Computational Methods

All molecular model calculations in this study were performed using the Gaussian 16 software package. Geometry optimizations (OPT) and electrostatic potential (ESP) analyses were carried out using the B3LYP functional combined with the 6-311G(d) basis set and empirical dispersion correction (EmpiricalDispersion = GD3).[5]

Periodic COF structures were simulated using density functional theory (DFT) as implemented in the Vienna Ab-initio Simulation Package (VASP). The Perdew–Burke–Ernzerhof (PBE) exchange-correlation functional was employed. The Brillouin zone was sampled using a 3 × 3 × 1 Γ-centered k-point mesh. Electron–ion interactions were described by the projector augmented wave (PAW) method, and the plane-wave energy cutoff was set to 500 eV. Exchange-correlation interactions were treated within the generalized gradient approximation (GGA) using the PBE functional. The electronic structure was further calculated using the HSE06 hybrid functional, which incorporates Hartree–Fock (HF) exchange with PBE exchange-correlation terms. The electronic band structure calculations were performed using VASP combined with the post-processing tool VASPKIT.[6]

Under alkaline conditions, the WOR on the COF surface can be divided into the following five stepwise one-electron oxidation processes, corresponding to the deprotonation of hydroxide or water molecules:

- **Step 1:** $^{*} + OH^{-} \rightarrow {}^{*}OH + e^{-}$
- **Step 2:** $^{*}OH + OH^{-} \rightarrow {}^{*}O + H_2O + e^{-}$
- **Step 3:** $^{*}O + OH^{-} \rightarrow {}^{*}O^{*}OH + e^{-}$
- **Step 4:** $^{*}O^{*}OH + OH^{-} \rightarrow {}^{*}OO^{*} + H_2O + e^{-}$
- **Step 5:** $OO \rightarrow {}^{*} + O_2$

For the ORR, the reaction pathway is as follows:

- **Step 6:** $O_2 + {}^{*} \rightarrow {}^{*}O_2$
- **Step 7:** $^{*}O_2 + H_2O + e^{-} \rightarrow {}^{*}OOH + OH^{-}$
- **Step 8:** $^{*}OOH + H_2O + e^{-} \rightarrow H_2O_2 + OH^{-} + {}^{*}$

Here, * represents an active site on the surface, and intermediates with “*” denote corresponding radical species adsorbed onto the surface.

S1.13 Gibbs Free Energy Calculations

To evaluate the thermodynamic feasibility of each reaction step, Gibbs free energy changes (ΔG) were calculated. The Gibbs free energy G(T) of each species was determined using the following equation:

$G(T)=E+H(T)-TS(T)$

where E is the electronic energy obtained from self-consistent field (SCF) calculations, and H(T) and S(T) represent the enthalpy and entropy at temperature T, respectively, accounting for thermodynamic contributions at finite temperature. For gas-phase species, vibrational, rotational, and translational contributions were included. For species adsorbed on the surface of COFs, only vibrational corrections were considered due to restricted rotational and translational degrees of freedom.

All calculations were conducted under standard conditions (298.15 K, pH = 12), and the effect of an external potential U = 1.23 V was incorporated. The pH correction term (ΔpH) was introduced as follows:

$$\Delta pH = -k_B T ln(10) \approx 0.71\ eV$$

Based on these considerations, the Gibbs free energy changes for the O_2 evolution pathway were calculated as follows:

- $\Delta G_1 = G_{*OH} - ½G_{H2} + \Delta G_{pH} + \Delta G_U$
- $\Delta G_2 = G_{*O} - G_{*OH} - ½G_{H2} + \Delta G_{pH} + \Delta G_U$
- $\Delta G_3 = G_{*O*OH} - G_{*O} - ½G_{H2} + \Delta G_{pH} + \Delta G_U$
- $\Delta G_4 = G_{O2} - G_{*OOH} - ½G_{H2} + \Delta G_{pH} + \Delta G_U$

where ΔG_U = –eU and U is the applied potential (1.23 V).

For the oxygen reduction pathway ($O_2 \rightarrow H_2O_2$), the Gibbs free energy changes of each elementary step are:

- $\Delta G_5 = G_{*O2} - G_{*} - G_{H2O} + G_{OH-} - eU + \Delta G_{pH}$
- $\Delta G_6 = G_{*OOH} - G_{*O2} - G_{H2O} + G_{OH-} - eU + \Delta G_{pH}$
- $\Delta G_7 = G_{H2O2} + G_{*} + G_{OH^-} G_{*OOH} - G_{H2O} - eU + \Delta G_{pH}$

To account for solvation effects, ab initio molecular dynamics (AIMD) simulations were carried out for the TP-AN-COF and TP-PZ-COF systems immersed in an aqueous environment containing 97 H_2O molecules.[7] The simulations were performed at 300 K to match the experimental temperature, using a time step of 1 ps in the canonical (NVT) ensemble with temperature controlled by Nosé–Hoover thermostats. Each AIMD trajectory was run for approximately 5500 ps, with the systems reaching thermal equilibrium after around 2000 ps.

S1.14 Synthesis of 2,7-diaminophenazine·benzylideneaniline (DAPH·Bnzph)

Based on a procedure reported in the literature.[8] In a 50 mL round-bottom flask, (±)-BINAP (94.0 mg, 0.15 mmol, 0.30 equiv.) and tris(dibenzylideneacetone)dipalladium(0) (92 mg, 0.05 mmol, 0.10 equiv.) were added to toluene (15 mL) under a nitrogen atmosphere. A mixture of solid sodium tert-butoxide (70 mg, 0.7 mmol, 1.4 equiv.), 2,7-dibromophenazine (170 mg, 0.5 mmol, 1.00 equiv.), and benzylideneaniline (0.5 mL, 1.00 mmol, 2.40 equiv.)

dissolved in toluene (25 mL) was then added to the flask. The reaction mixture was heated to 110 °C and stirred under reflux for 16 hours. After cooling to room temperature, the toluene was removed under reduced pressure. Ethanol (~100 mL) was added to the residue and stirred at ~80 °C for 30 minutes. The resulting solid was collected by hot filtration to afford crude DAPH·Bnzph as a yellow solid (crude ^{1}H NMR purity >90%).

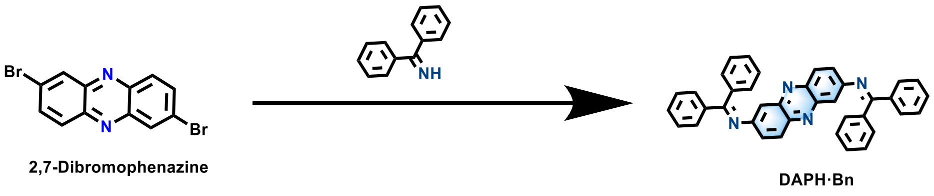

S1.15 Synthesis of TP-PZ COF

In a 10 mL sealed pressure tube, TFP (6.30 mg, 0.03 mmol) and 2,7-diphenylphenazine benzophenone imine (DAPH·Bnzph, 10.77 mg, 0.02 mmol) were added, followed by 1 mL of a mixed solvent system of mesitylene and 1,4-dioxane (v/v = 1:1). The tube was sealed and sonicated at room temperature for 10 minutes. Then, 0.1 mL of 6 M acetic acid (ice-cold) was added directly to the reaction mixture, and the mixture was degassed by three freeze–pump–thaw cycles. The tube was wrapped in aluminum foil to prevent potential side reactions induced by benzophenone photoreactivity, and then placed in a preheated oil bath at 120 °C without stirring for 3 days. After cooling to room temperature, the reaction mixture was filtered through a Buchner funnel lined with filter paper, rinsing the tube thoroughly with acetone to ensure complete transfer of the product. The collected solid was successively purified by stirring in hot DMF (100 mL, 90 °C, 30 min), followed by hot filtration. This washing step was repeated with hot DMF (100 mL, 90 °C, 30 min), anhydrous ethanol (100 mL, 80 °C, 30 min), and finally acetone (100 mL, 60 °C, 30 min). The final solid was collected by filtration and transferred to a ceramic crucible for drying under vacuum (~20 mTorr) at 120 °C for 24 hours. The obtained TP-PZ covalent organic framework was a red-purple solid.

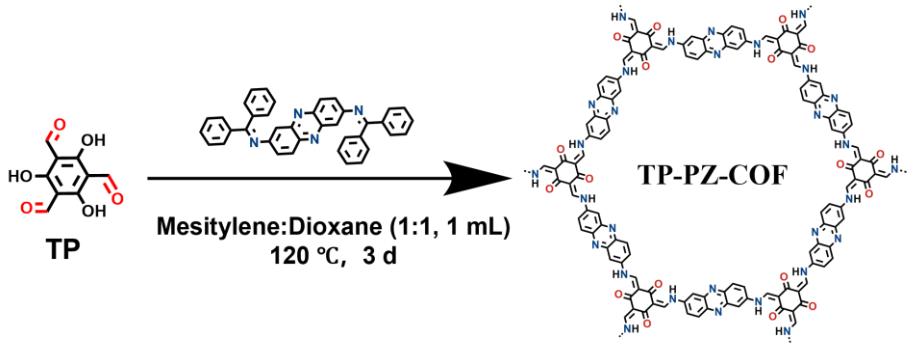

S1.16 Synthesis of Tp-AN-COF

In a 10 mL sealed pressure tube, TFP (8.40 mg, 0.04 mmol) and 2,7-diaminoanthracene (14.49 mg, 0.06 mmol) were added, followed by 1 mL of a mixed solvent of mesitylene and 1,4-dioxane (v/v = 1:1). The tube was sealed and sonicated at room temperature for 10 minutes. Then, 0.1 mL of 6 M acetic aci+d (ice-cold) was added directly to the mixture, which was subsequently degassed via three freeze–pump–thaw cycles. The reaction vessel was then placed in a preheated oil bath at 120 °C without stirring for 3 days. After cooling to room temperature, a red solid was obtained.

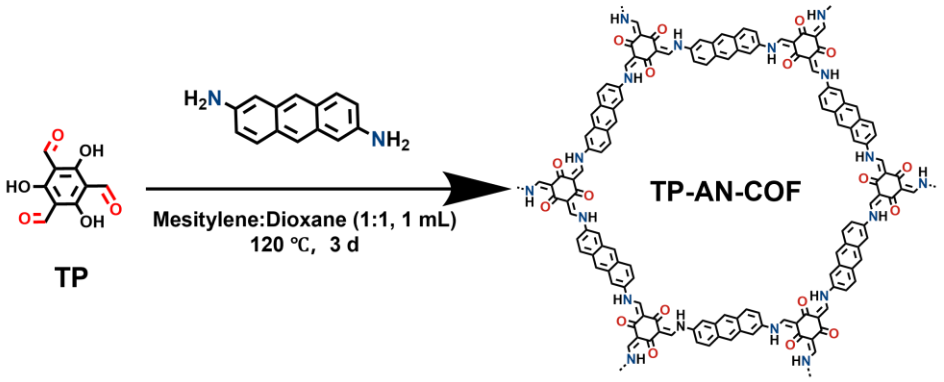

Section S2. Characterization Fig.s

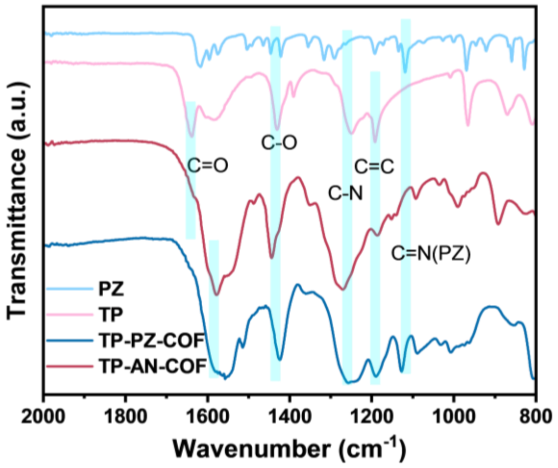

Fig. S3 FT-IR spectra of TP-PZ-COF, TP-AN-COF and precursors.

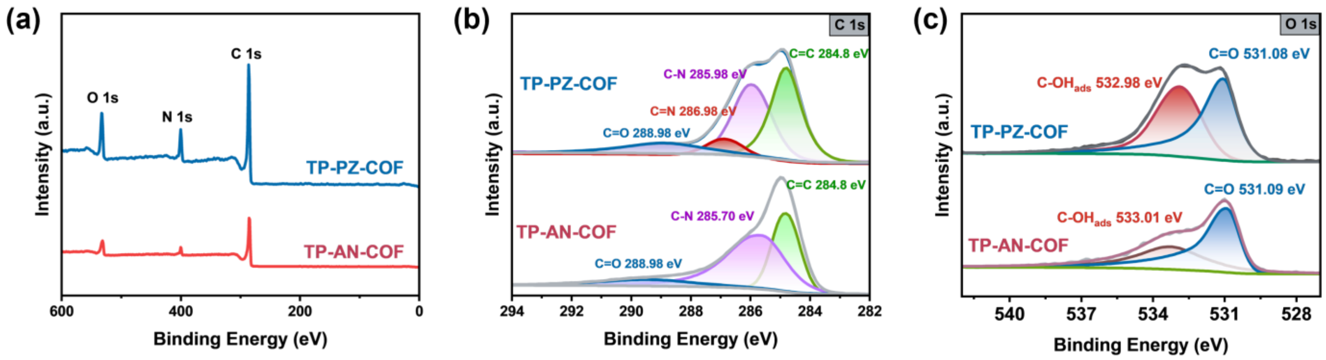

Fig. S4 (a) XPS survey spectra for TP-PZ-COF and TP-AN-COF; (b) C 1s XPS spectra for TP-PZ-COF and TP-AN-COF; (c) O 1s XPS spectra for TP-PZ-COF and TP-AN-COF.

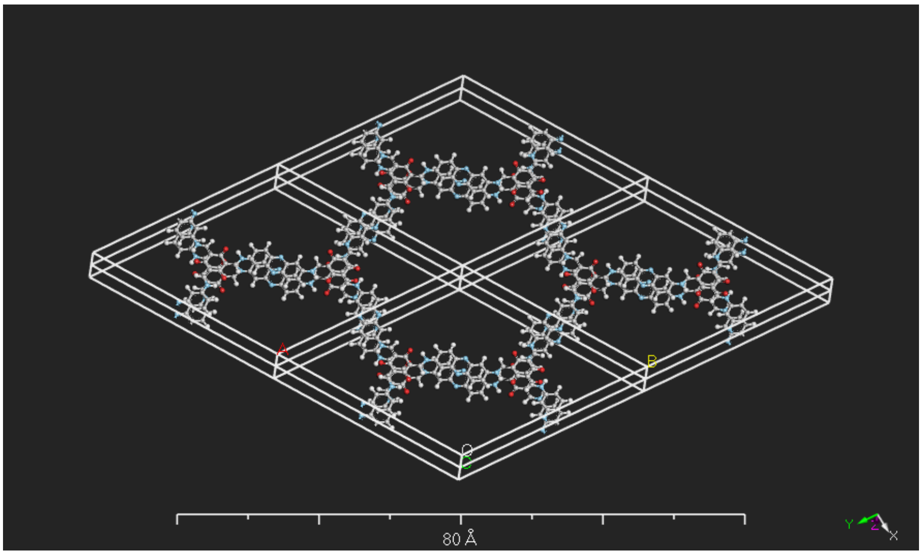

Fig. S5 The reconstructed lattice structure of TP-PZ-COF via the simulated AA stacking model.

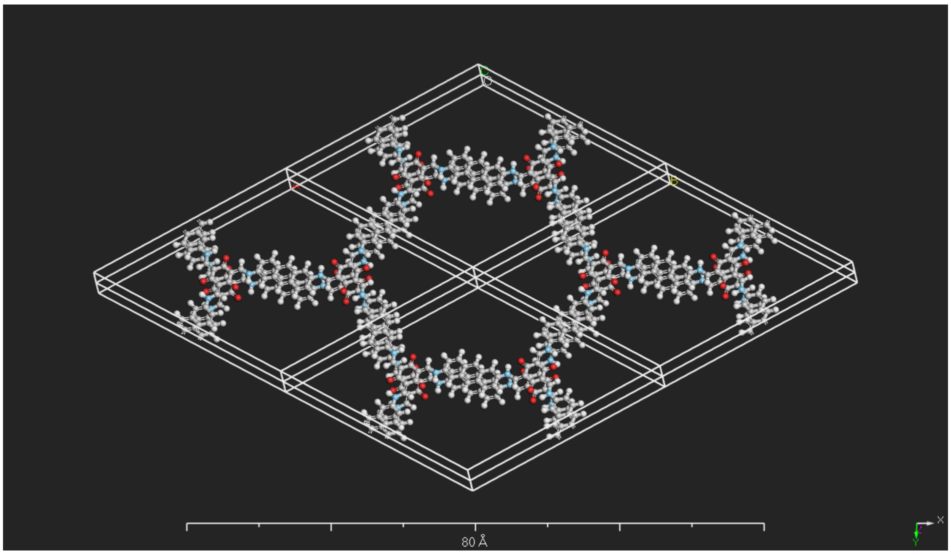

Fig. S6 The reconstructed lattice structure of TP-AN-COF via the simulated AA stacking model.

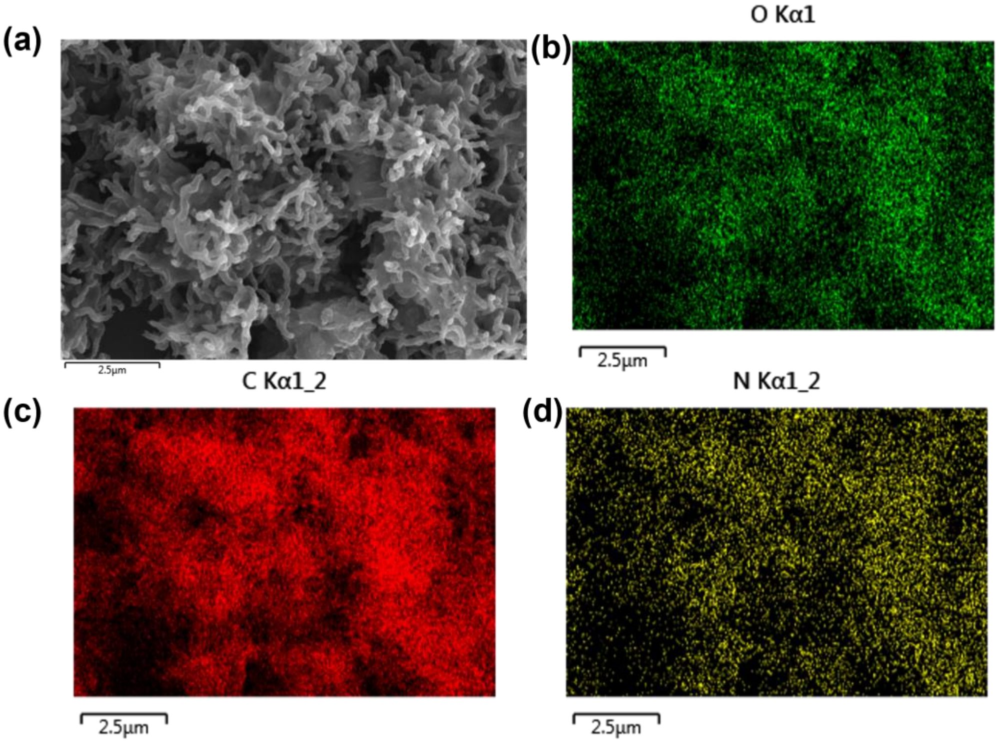

Fig. S7 (a) SEM images and (b), (c), (d)EDS of TP-PZ-COF.

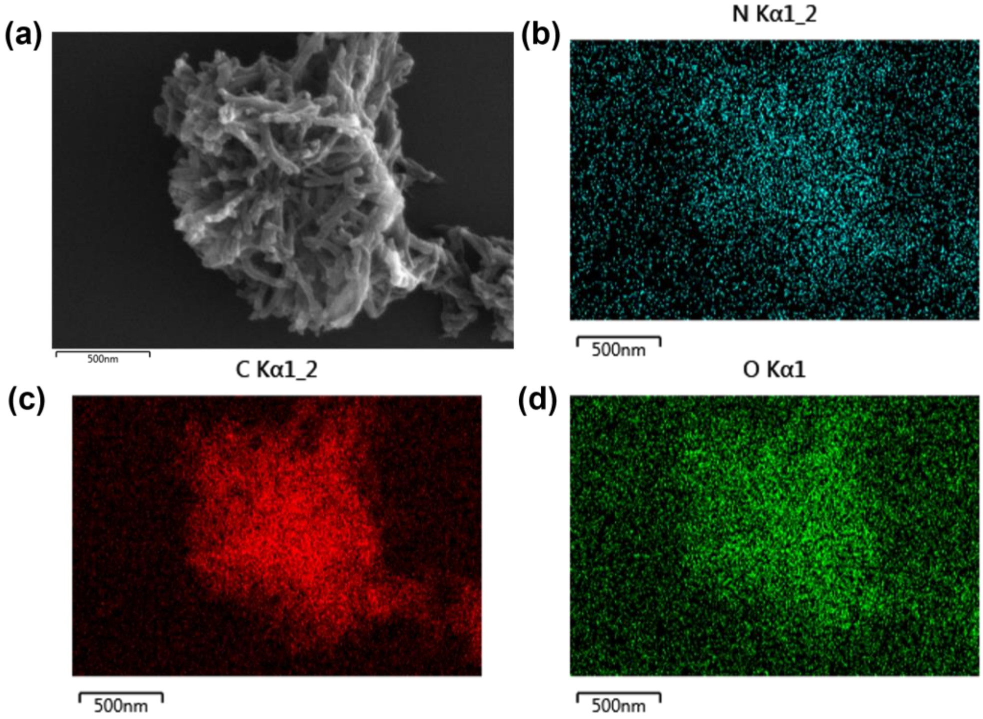

Fig. S8 (a) SEM images and (b), (c), (d)EDS of TP-AN-COF.

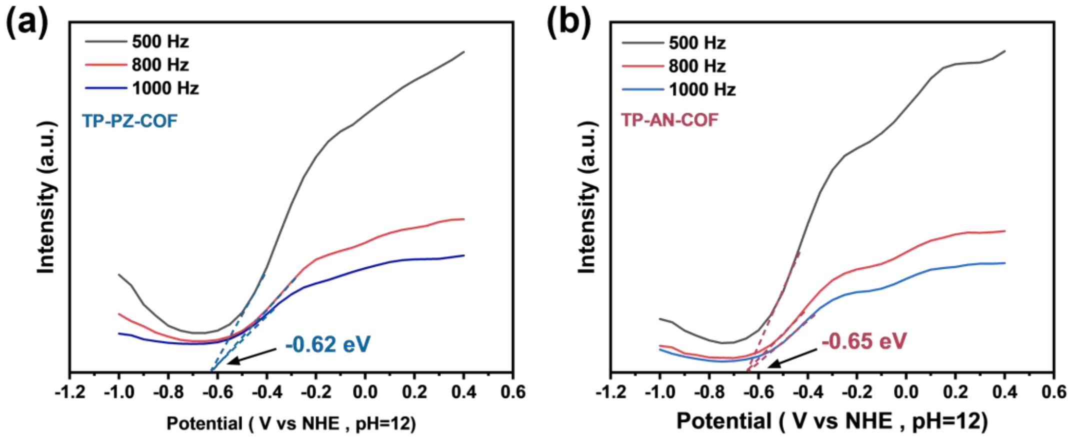

Fig. S9 Mott-Schottky measurements of (a) TP-PZ-COF and (b) TP-AN-COF in 0.01 M NaOH.

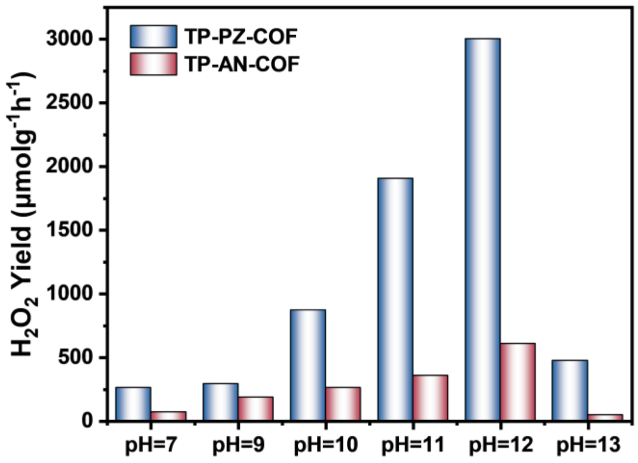

Fig. S10 H_2O_2 production yields under alkaline conditions (air atmosphere) at varying pH values of TP-PZ-COF and TP-AN-COF.

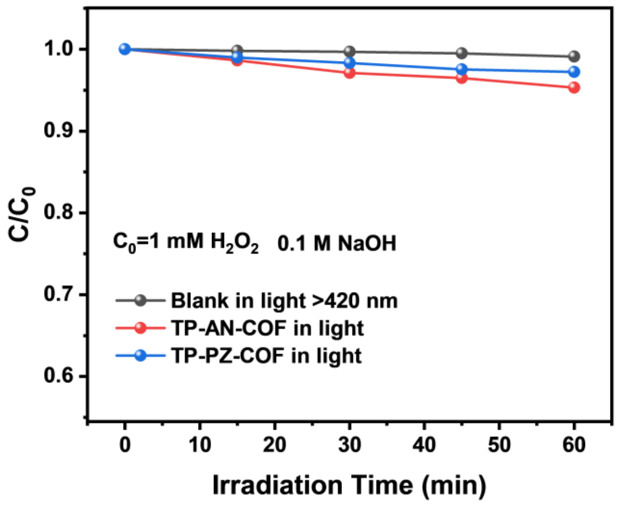

Fig. S11 Decomposition of H_2O_2 over TP-PZ-COF and TP-AN-COF in N_2 atmosphere (λ > 420nm, 300 W Xenon lamp; 5.0 mg catalyst in 50 mL 1 mM H_2O_2 solution with 0.01 M NaOH, 15 °C).

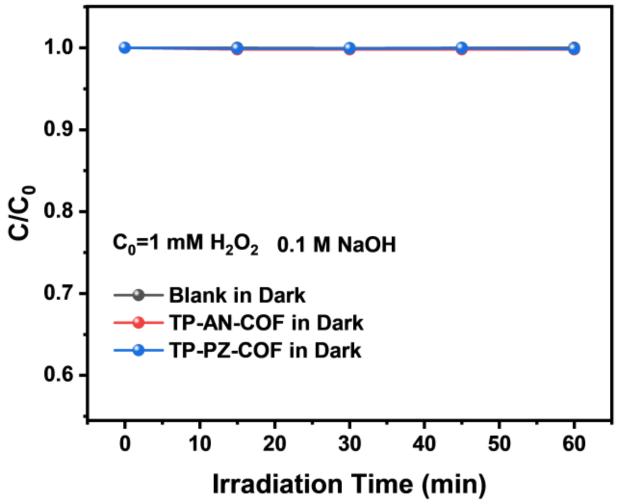

Fig. S12 Decomposition of H_2O_2 over TP-PZ-COF and TP-AN-COF in Dark (5.0 mg catalyst in 50 mL 1 mM H_2O_2 solution with 0.01 M NaOH, 15 °C).

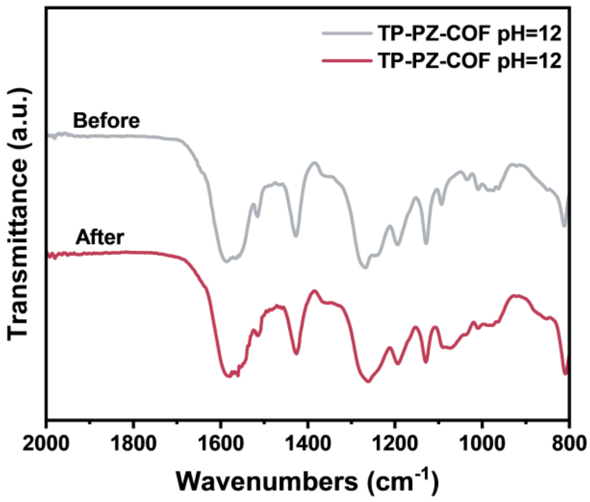

Fig. S13 FTIR spectra of TP-PZ-COF before and after photocatalytic cycles in alkaline water.

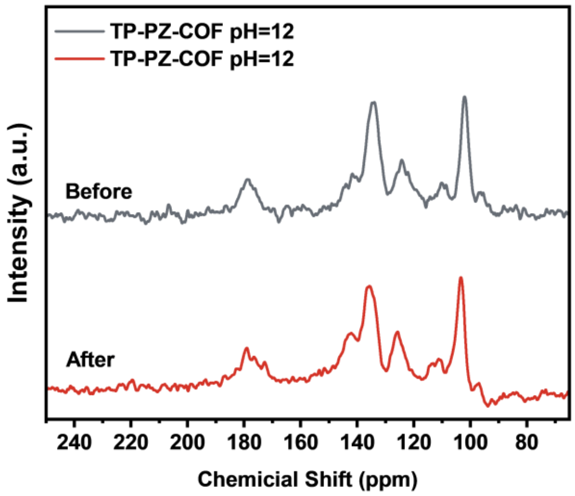

Fig. S14 ^{13}C NMR spectra of TP-PZ-COF before and after photocatalytic cycles in alkaline water.

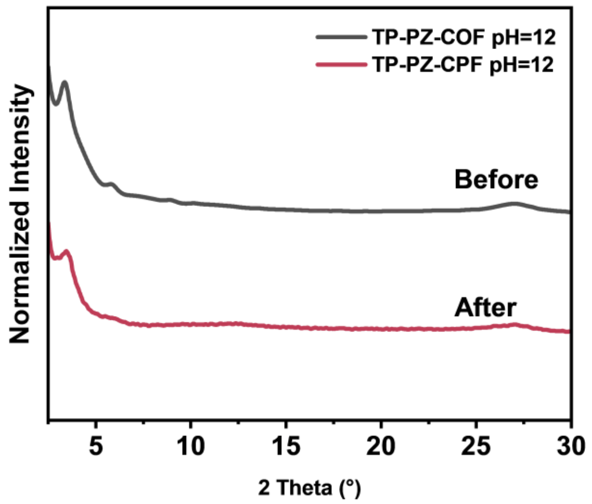

Fig. S15 PXRD spectra of TP-PZ-COF before and after photocatalytic cycles in alkaline water.

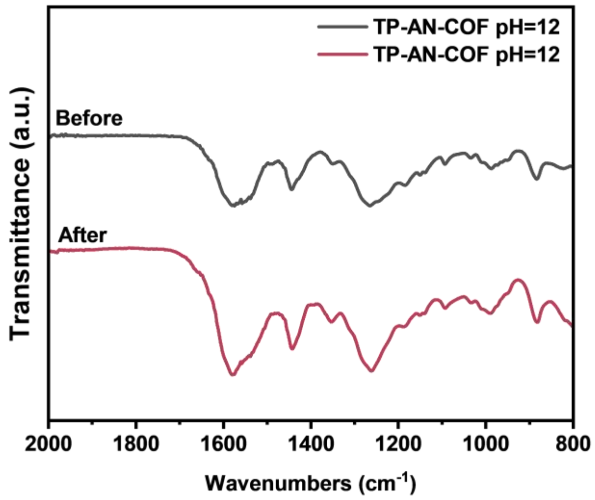

Fig. S16 FTIR spectra of TP-AN-COF before and after photocatalytic cycles in alkaline water.

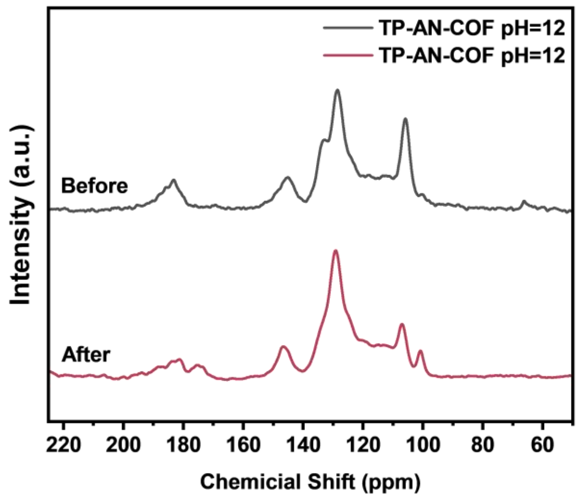

Fig. S17 ^{13}C NMR spectra of TP-AN-COF before and after photocatalytic cycles in alkaline water.

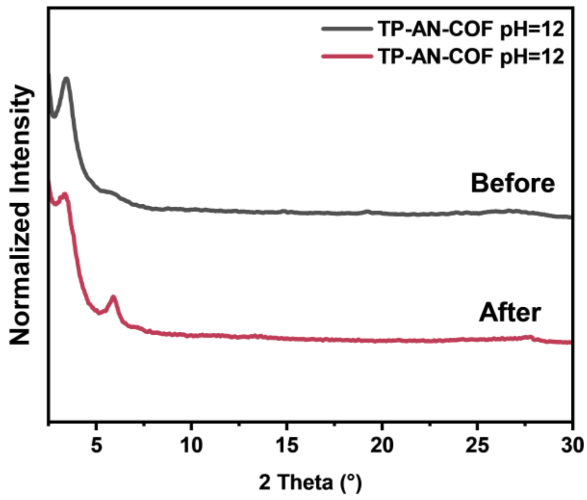

Fig. S18 PXRD spectra of TP-AN-COF before and after photocatalytic cycles in alkaline water.

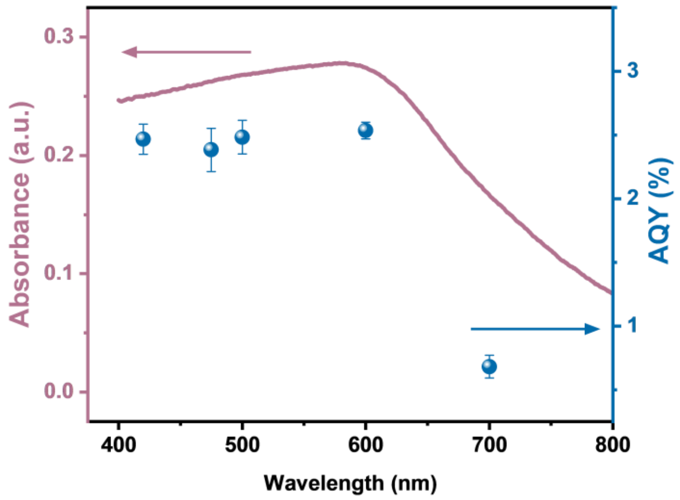

Fig. S19 UV/vis DRS spectrum and AQY comparison of TP-PZ-COF.

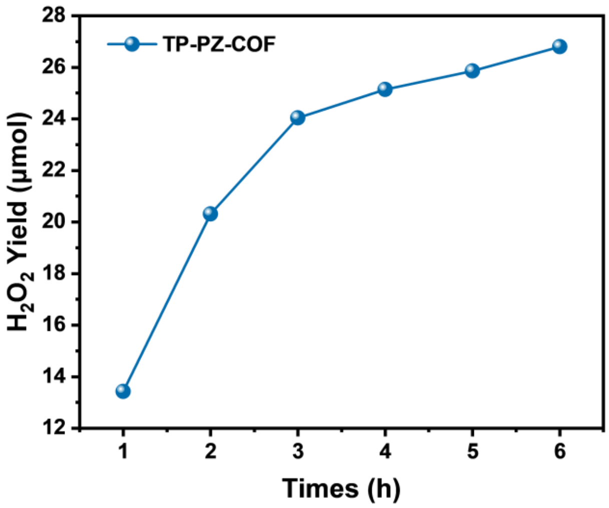

Fig. S20 Long-term continuous H_2O_2 photosynthesis experiment of TP-PZ-COF.

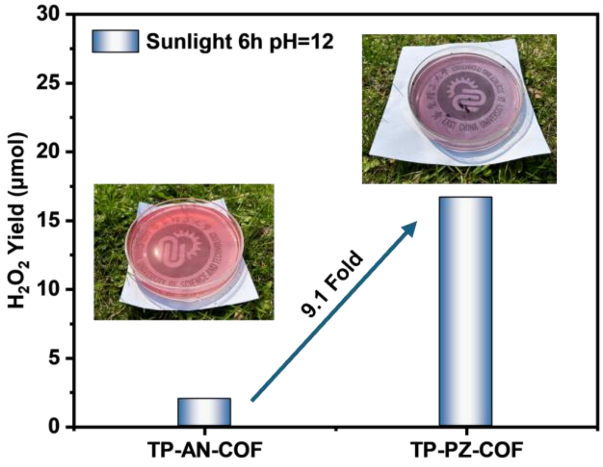

Fig. S21 Comparison of H_2O_2 production by TP-PZ-COF and TP-AN-COF under natural light irradiation for 6 h.

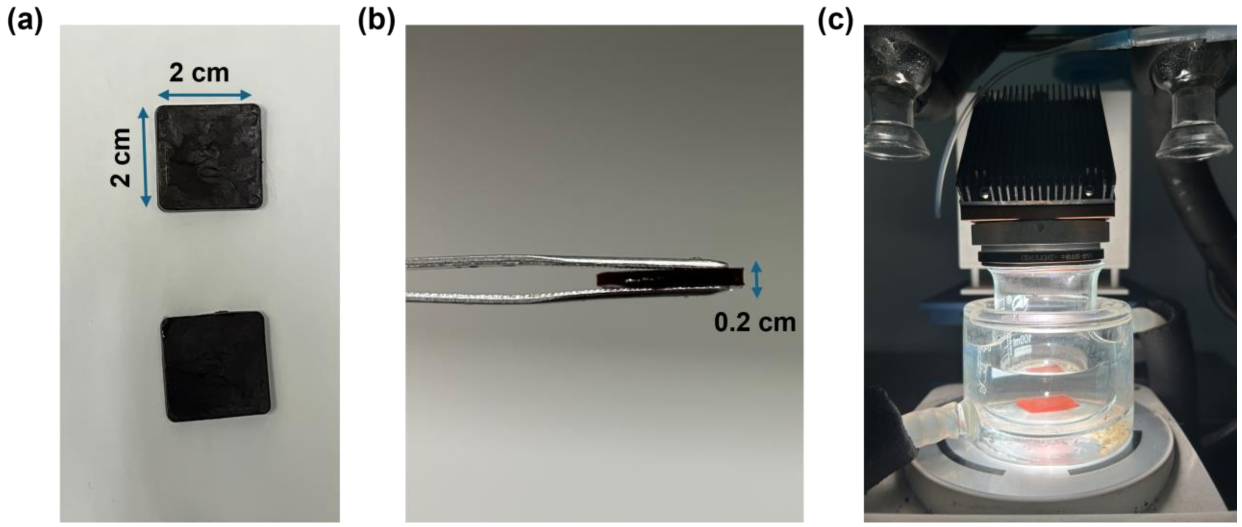

Fig. S22 (a) Photograph of the TP-PZ-COF-doped polyacrylamide (PAAm) hydrogel film with 2 cm × 2 cm.(b) Cross-sectional view showing a film thickness of approximately 0.1 cm.(c) Schematic illustration of the photocatalytic H_2O_2 generation under simulated sunlight irradiation.

To prepare the TP-PZ-COF polyacrylamide hydrogel membrane, 1 g of acrylamide monomer was added to a small glass vial, followed by sequential addition of ammonium persulfate (40.0 mg), N, N′-methylenebisacrylamide (20.0 mg), and TP-PZ-COF powder (20.0 mg). The mixture was then dispersed in 4 mL of deionized water with sonication for 30 minutes. Subsequently, 1 mL of the resulting dispersion was transferred into a square mold (2 cm × 2 cm), and 0.5 μL of N, N, N′, N′-tetramethyl ethylenediamine (TEMED) was added to initiate polymerization. The mixture was left undisturbed at 25 °C for 10 minutes to form a self-standing membrane via in situ gelation. Under simulated solar irradiation at 25 °C in alkaline aqueous solution, the resulting COF hydrogel membrane achieved a remarkable photocatalytic H_2O_2 production rate of up to 55.4 mmolh^{-1} m^{-2}.

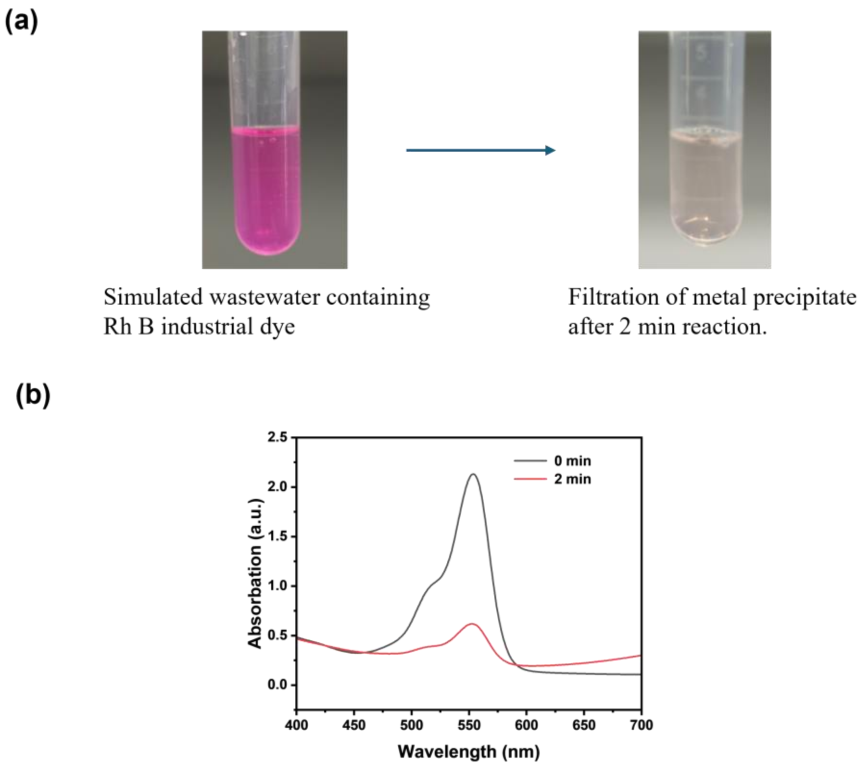

Fig. S23 (a) Simulated Rh B industrial wastewater treated with the solution obtained after 6 h irradiation of TP-PZ-COF-PAM hydrogel film. (b) UV–Vis absorption spectra of Rh B at 0 min and 2 min

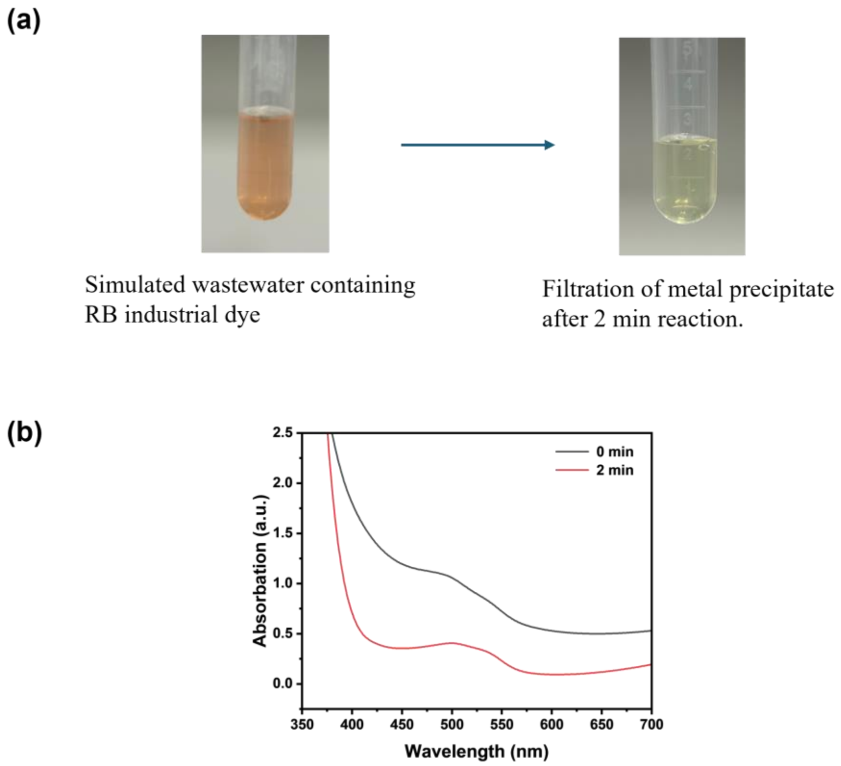

Fig. S24 (a) Simulated RB industrial wastewater treated with the solution obtained after 6 h irradiation of TP-PZ-COF–PAM hydrogel film. (b) UV–Vis absorption spectra of RB at 0 min and 2 min.

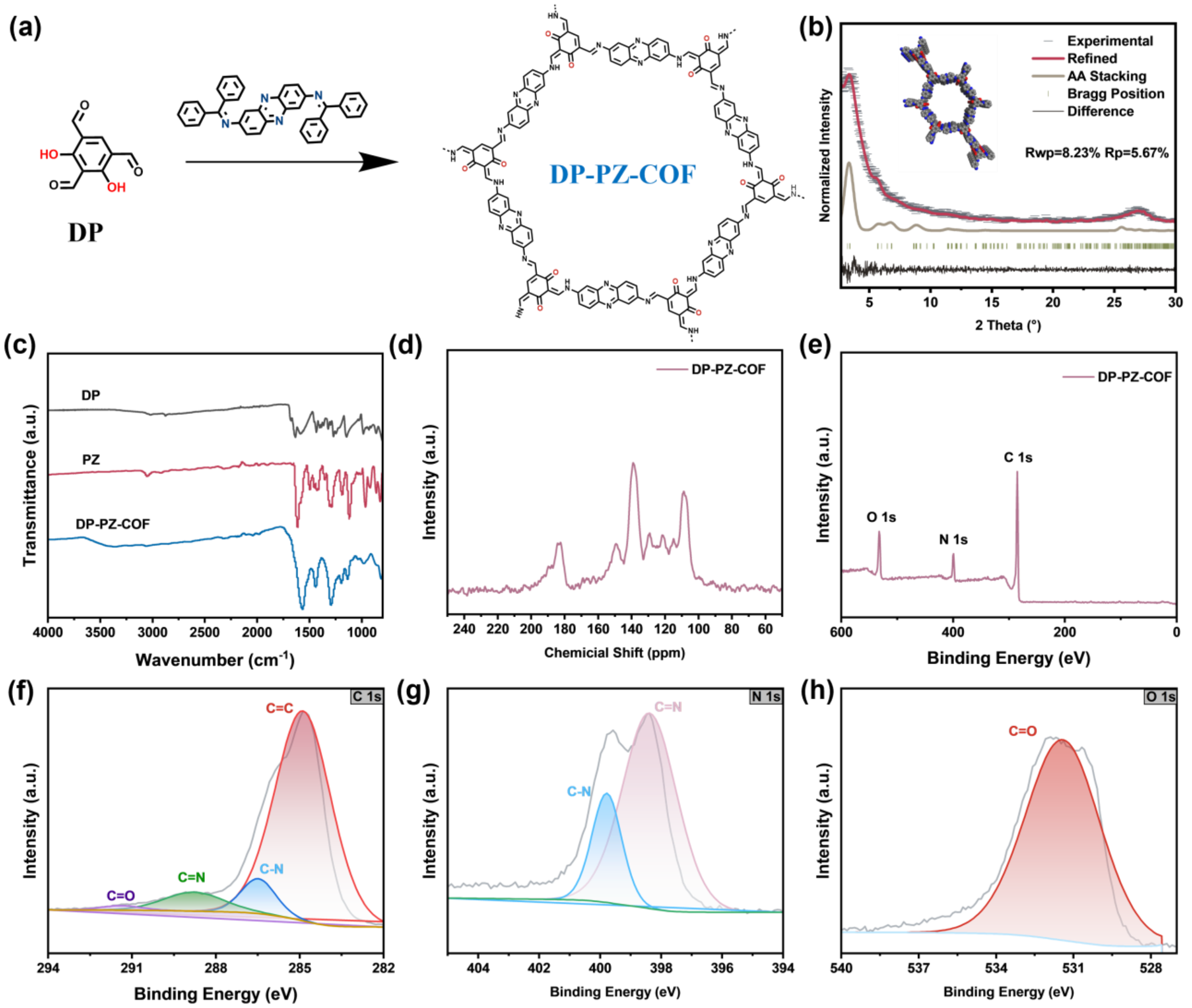

Fig. S25 (a-b) Synthesis of DP-PZ-COF and its structural characterization by powder X-ray diffraction (PXRD), (c) Fourier-transform infrared (FT-IR) spectroscopy, (d) solid-state ^{13}C NMR spectroscopy, (e-h) X-ray photoelectron spectroscopy (XPS).

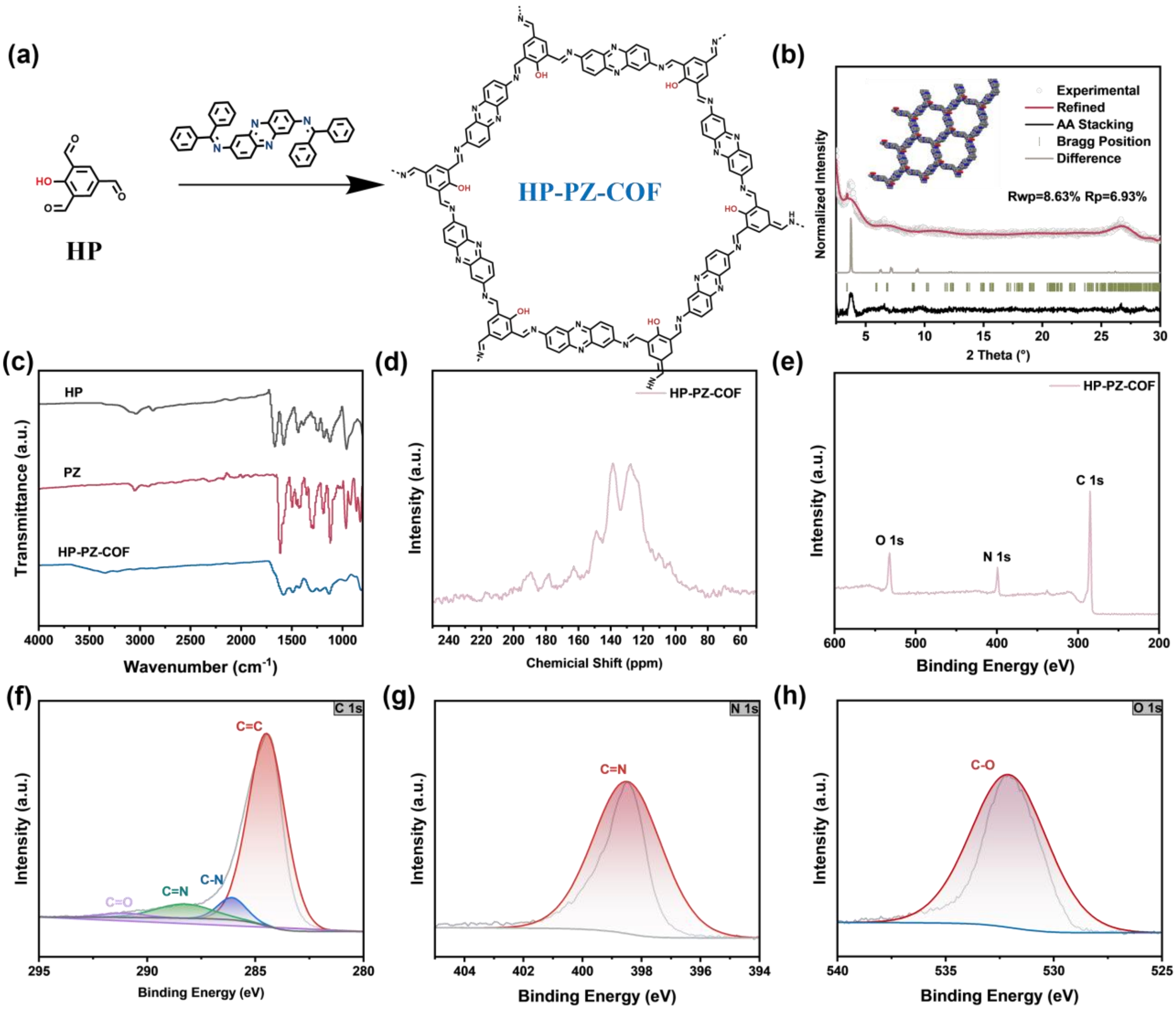

Fig. S26 (a-b) Synthesis of HP-PZ-COF and its structural characterization by powder X-ray diffraction (PXRD), (c) Fourier-transform infrared (FT-IR) spectroscopy, (d) solid-state ^{13}C NMR spectroscopy, (e-h) X-ray photoelectron spectroscopy (XPS).

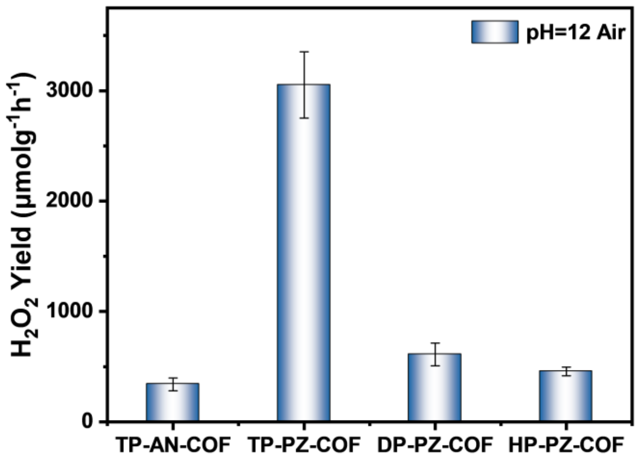

Fig. S27 Photocatalytic H_2O_2 generation rates of TP-AN-COF, TP-PZ-COF, DP-PZ-COF, and HP-PZ-COF under air at pH=12.

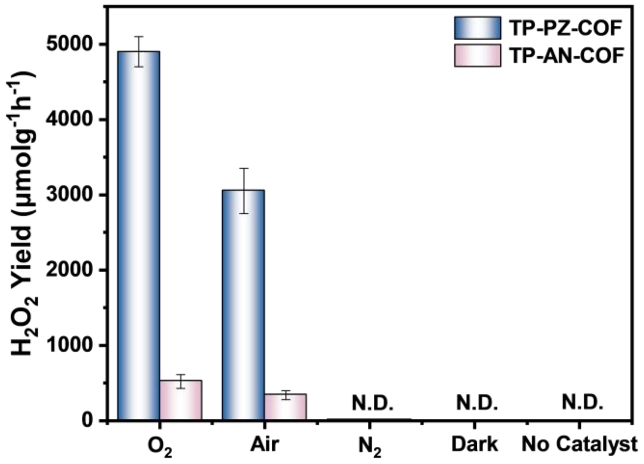

Fig. S28 Comparison of H_2O_2 production of TP-PZ-COF and TP-AN-COF under different conditions.

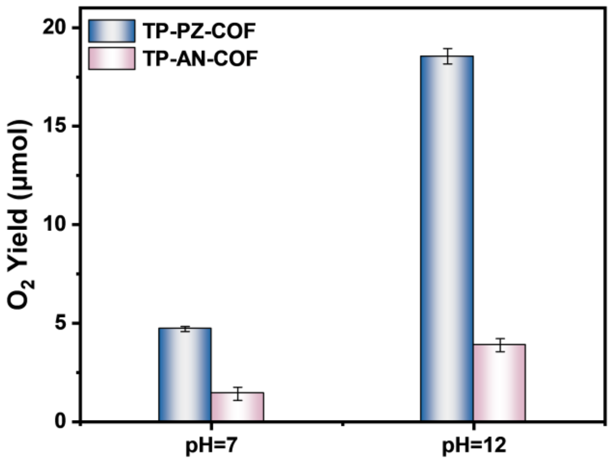

Fig. S29 Comparison of O_2 evolution over TP-PZ-COF and TP-AN-COF at pH = 7 and pH = 12 within 1 h using $K_2Cr_2O_7$ as electron scavenger.

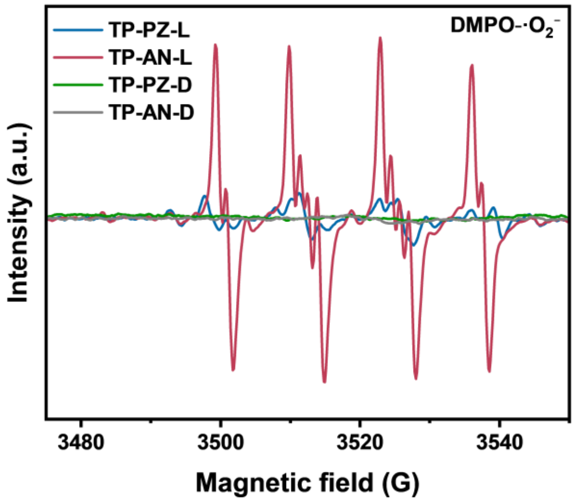

Fig. S30 DMPO-•O_2^- EPR spectra of TP-PZ-COF and TP-AN-COF under dark and light irradiation.

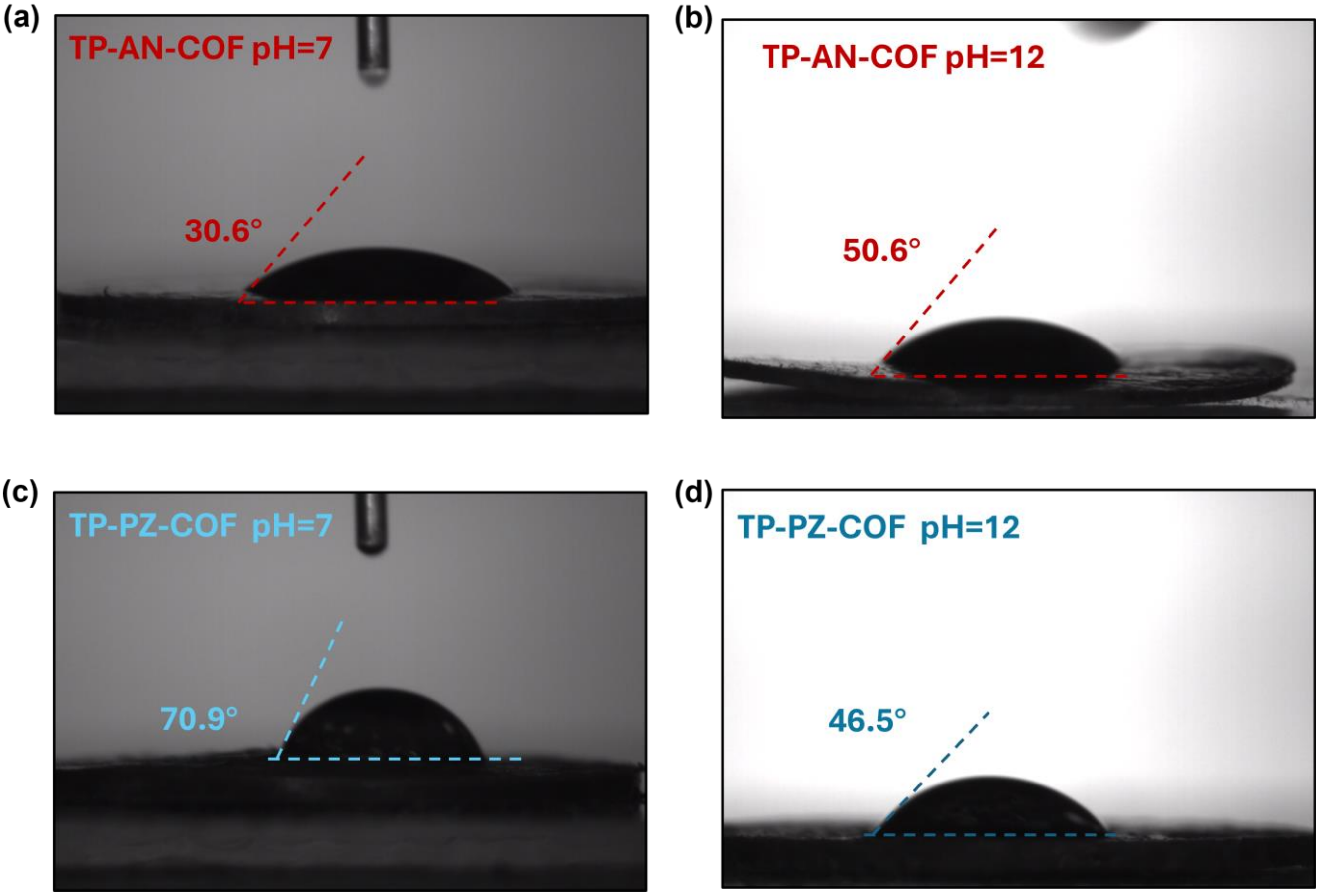

Fig. S31 (a)Contact angle measurements of TP-AN-COF under pH=7 conditions. (b)Contact angle measurements of TP-AN-COF under pH=12 conditions. (c) Contact angle measurements of TP-PZ-COF under pH=7 conditions. (d) Contact angle measurements of TP-PZ-COF under pH=12 conditions.

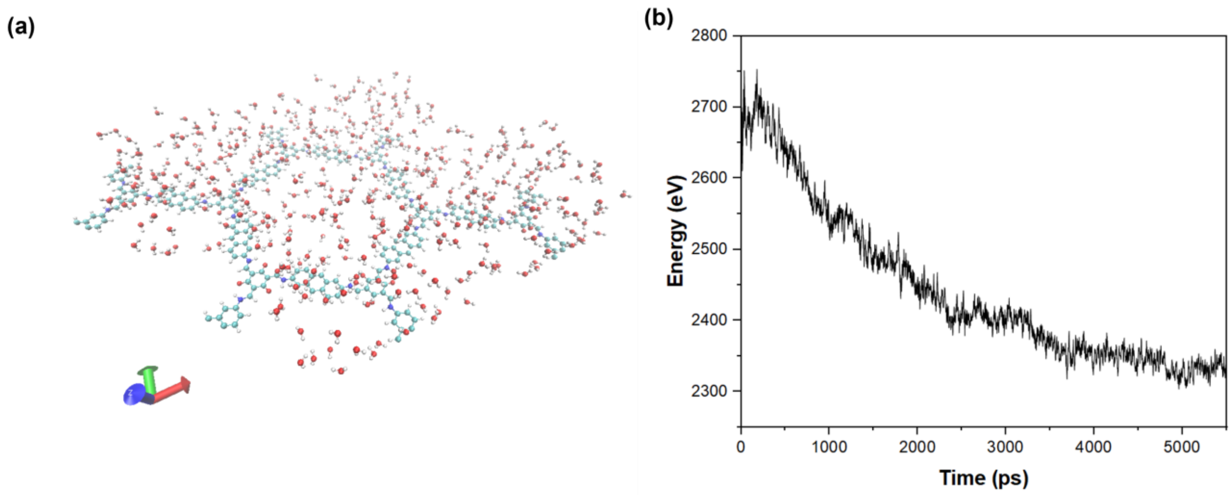

Fig. S32 (a) Water distribution model and (b)energy profile of TP-AN-COF during 5.5 ns AIMD simulation.

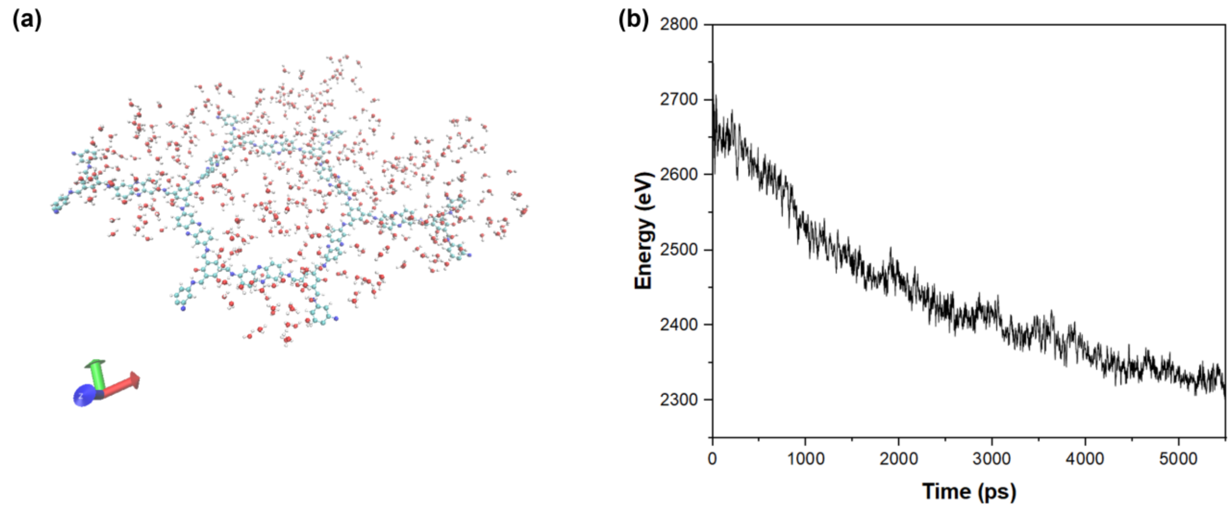

Fig. S33 (a) Water distribution model and (b)energy profile of TP-PZ-COF during 5.5 ns AIMD simulation.

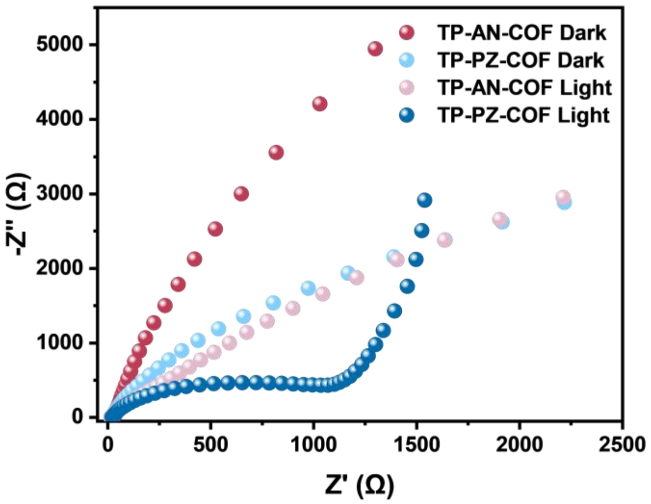

Fig. S34 EIS Nyquist plots of TP-PZ-COF and TP-AN-COF under dark and visible light irradiation (λ>420 nm, 300 W Xe lamp) in alkaline solution.

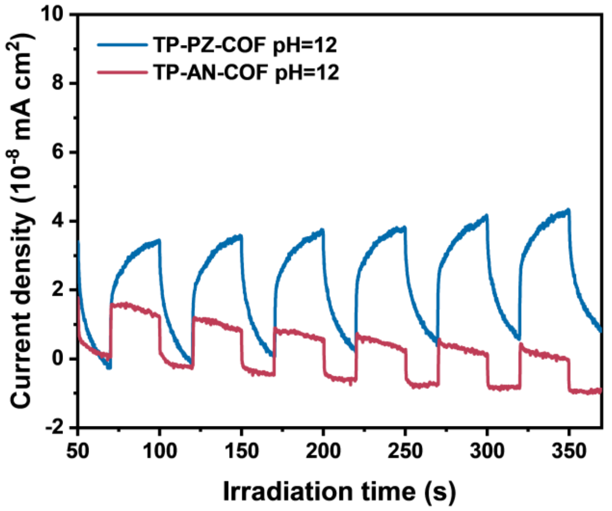

Fig. S35 Transient photocurrent responses of TP-PZ-COF and TP-AN-COF under visible light irradiation (λ > 420 nm, 300 W Xe lamp) in alkaline solution.

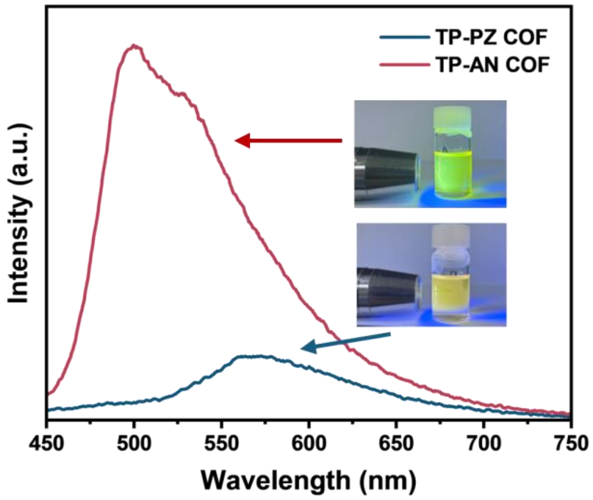

Fig. S36 Fluorescence intensity of TP-PZ-COF and TP-AN-COF dispersed in N-Methyl-2-pyrrolidone (NMP) solution.

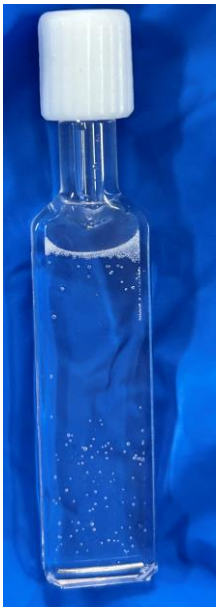

Fig. S37 Photograph of the reaction vessel after 30 min of 420 nm laser irradiation on TP-PZ-COF.

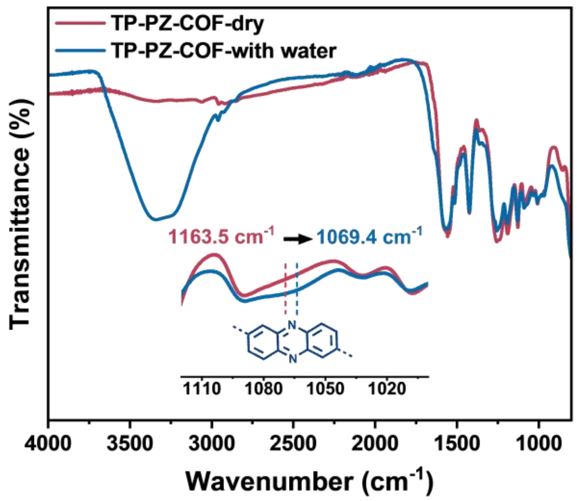

Fig. S38 FTIR spectra of TP-PZ-COF before and after water immersion.

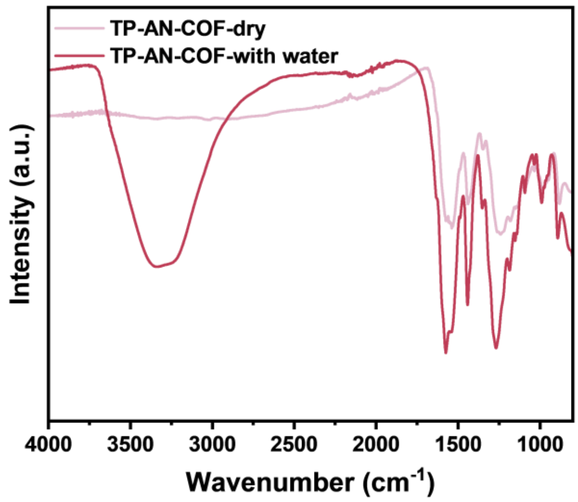

Fig. S39 FTIR spectra of TP-AN-COF before and after water immersion.

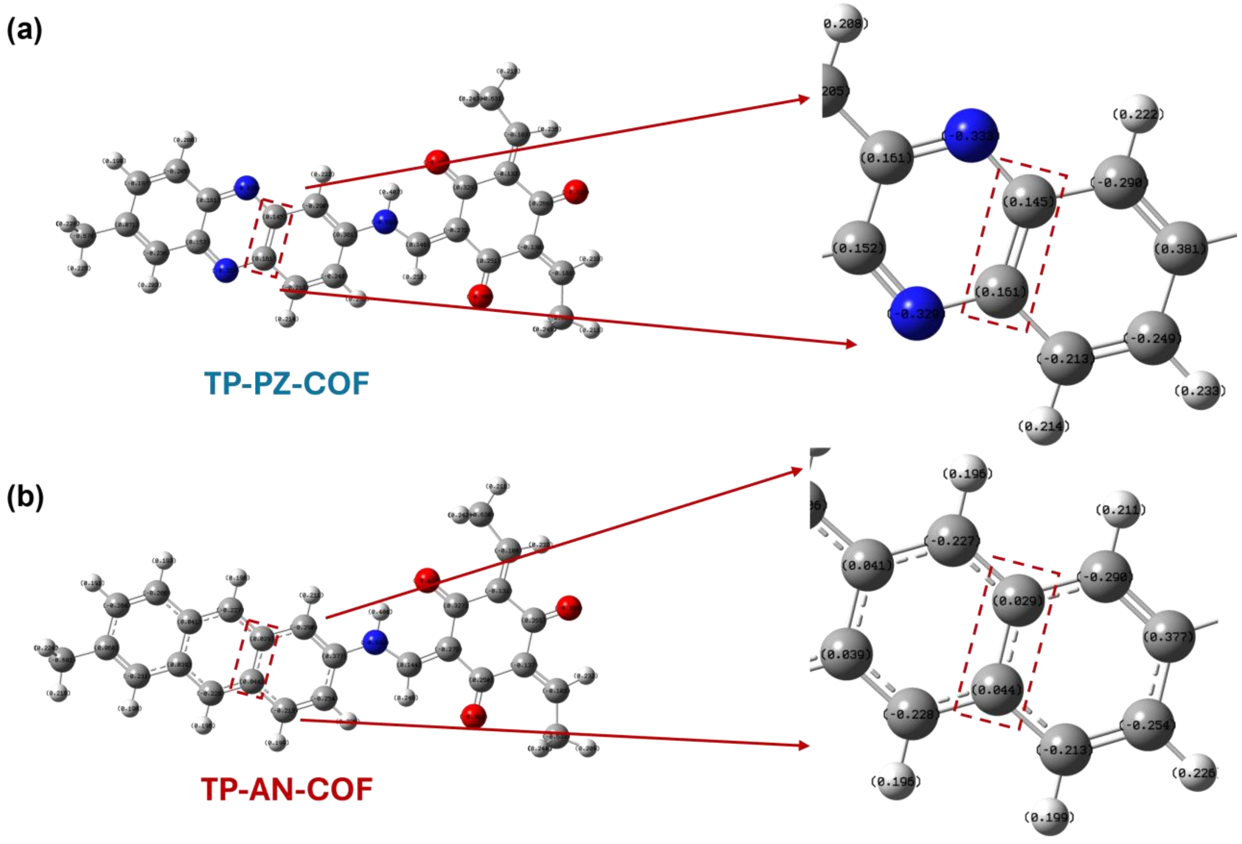

Fig. S40 Charge density distribution maps of (a)TP-PZ-COF and (b)TP-AN-COF.

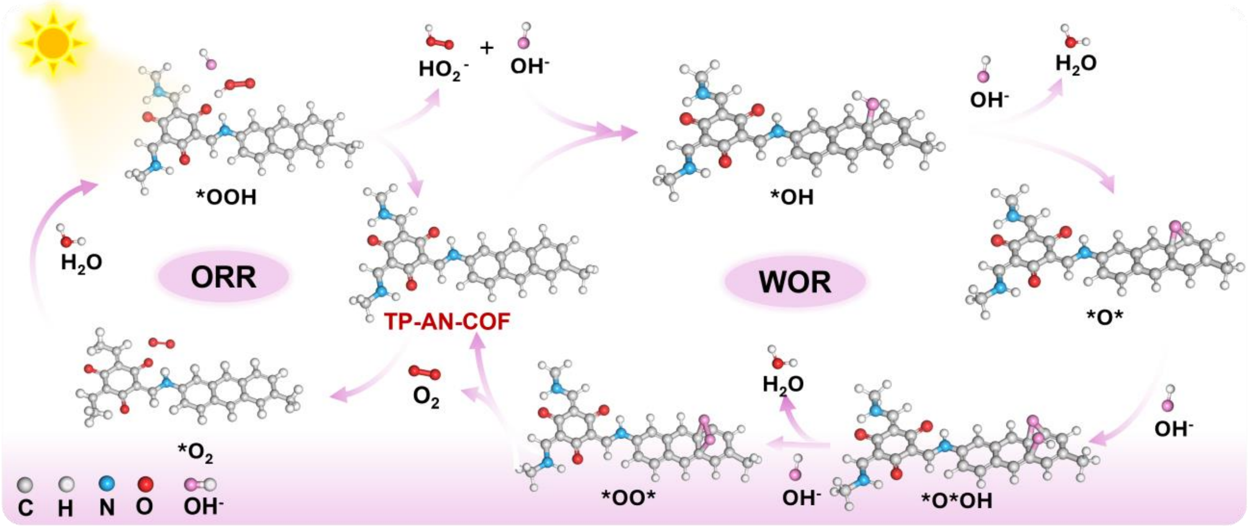

Fig. S41 Schematic illustration of the overall H_2O_2 production mechanism over TP-AN-COF.

Section S3. Supplementary Tables

Table S1. Fractional atomic coordinates and the unit cell of TP-AN-COF

TP-AN-COF		Space group = *P6*<br>a = 30.4841 Å, b = 30.4841 Å, c = 3.7236 Å,<br>α = 90.0000°, β = 90.0000°, γ = 120.0000°	
C1	C	0.41197	0.8604
C2	C	0.37708	0.87872
C3	C	0.39378	0.93098
C4	C	0.44666	0.96448
C5	C	0.48248	0.94663
C6	C	0.46488	0.89418
C7	C	0.46493	0.01999
N8	N	0.39925	0.81115
C9	C	0.35384	0.76827
C10	C	0.34461	0.71805
C11	C	0.38418	0.70635
O12	O	0.68766	0.42941
H13	H	0.33458	0.85085
H14	H	0.36501	0.94448
H15	H	0.49306	0.87994
H16	H	0.43458	0.80907

Table S2. Fractional atomic coordinates and the unit cell of TP-PZ-COF

TP-PZ-COF		Space group = *P6*<br>a = 30.1542 Å, b = 30.1542 Å, c = 3.7431 Å,<br>α = 90.0000°, β = 90.0000°, γ = 120.0000°	
C1	C	0.1377	0.59547
C2	C	0.48794	0.11563
C3	C	0.4546	0.06189
C4	C	0.478	0.03113
C5	C	0.53258	0.05248
C6	C	0.56542	0.10624
N7	N	0.44778	0.97937
N8	N	0.43202	0.81033
C9	C	0.38135	0.7756
C10	C	0.35839	0.7215
C11	C	0.30396	0.69142
O12	O	0.7135	0.43566
H13	H	0.4703	0.1414
H14	H	0.41136	0.04547
H15	H	0.60872	0.1231
H16	H	0.45346	0.78926

Table S3. Comparison of photocatalytic performances among recently reported COFs

T	Photocatalysts	Reaction solution	pH	H_2O_2 generation rate [μmolh^{-1}g^{-1}]	AQY	SCC (298K)	Ref.
1	TP-PZ-COF	H_2O/O_2	12	4961	2.53% (600nm)	0.72%	This work
2	CityU-45	H_2O/O_2	13	4854	-	-	*Angew. Chem. Int. Ed.* 2025, e22298.
3	COF-TPT-Azo	H_2O	11	1498	-	-	*Angew. Chem. Int. Ed.* 2024, 63, e202409250.
4	Por-BQ-COF	H_2O	12	1525	5.05% (420nm)	-	*Adv. Mater.* 2025, 37, 2415126.
5	Kf-AQ	H_2O/O_2	13	4784	15.8% (400nm)	0.7%	*Nat. Commun.* 2024,15, 2649.
6	COF-TfpBpy	H_2O/Air	7	1042 (μM h^{-1})	8.1%	0.57%	*Angew. Chem. Int. Ed.* 2022, 61, e202200413.
7	HEP-TAPT-COF	H_2O/O_2	7	87.50 (μmolh^{-1})	-	0.65%	Angew. Chem. Int. Ed. 2023, 62, e202217479.
8	Bpy-TAPT	H_2O/O_2	7	4038	8.6% (420nm)	-	*Appl. Catal. B Environ.* 2023, 331, 122691.
9	FS-COFs	H_2O/Air	7	3904.2	6.21% (420nm)	-	*Angew. Chem. Int. Ed.* 2023, 62, e202305355.
10	TD-COF/TT-COF	H_2O/O_2	7	4620/ 4245	-	0.15%/ 0.14%	*Angew. Chem. Int. Ed.* 2023, 62, e202309624.
11	TZ-COF	H_2O	7	268	0.6% (475nm)	0.036	*Angew. Chem. Int. Ed.* 2023, 62, e202309480.
12	COF-JLU51	H_2O/O_2	7	4200	6.4% (420nm)	0.19%	*Angew. Chem. Int. Ed.* 2024, 63, e202411546.
13	COF-N32	H_2O	7	605	6.2% (459nm)	0.31%	*Nat. Commun.* 2023, 14, 4344.
14	COF-2CN	H_2O/O_2	7	1601	6.8% (459nm)	0.6%	*Angew. Chem. Int. Ed.* 2024, 63, e202318562.
15	FS-OHOMe-COF	H_2O/O_2	7	1.0 (mMh^{-1})	-	0.58%	*Angew. Chem. Int. Ed.* 2024, 63, e202403926.

16	o-COF-TpPzda	H_2O/O_2	7	4396	-	0.46%	*Angew. Chem. Int. Ed.* 2024, 63, e202404077.
17	PyIm-COF	H_2O/O_2	7	5850	3.70% (420nm)	0.28%	*Angew. Chem. Int. Ed.* 2024, 63, e202404563.
18	CHF-DPDA	H_2O/O_2	7	69 (μmol h^{-1})	-	0.78%	*Adv. Mater.* 2022, 34, e202107480.
19	TTF-BT-COF	H_2O/O_2	7	1380 (μMh^{-1})	11.19% (420nm)	0.49%	*Angew. Chem. Int. Ed.* 2023, 62, e202218868.
20	Bpt-CTF	H_2O/O_2	7	3268.1	8.6% (400nm)	-	*Adv. Mater.* 2022, 34, e2110266.
21	PD_2^+-$COF_{16.7}$	H_2O	7	1732	-	0.34%	*Angew. Chem. Int. Ed.* 2023, 62, e202315456.
22	QP-HPTP-COF	H_2O/O_2	7	4388	-	1.41%	*Adv. Mater.* 2025, 37, 2410247.
23	CTF-NSs	H_2O/O_2	7	5007	16.8% (420nm)	0.91%	*J. Am. Chem. Soc.* 2024, 146, 29943−29954.
24	TBA-COF	H_2O/O_2	7	8880	-	0.91%	*Adv. Funct. Mater.* 2025, 35, 2421514.
25	EBBT-COF	H_2O/O_2	7	5690	15.14% (420nm)	1.17%	*Adv. Energy Mater*. 2025, 2404497.
26	TB-TT	H_2O/O_2	7	3550	10.13% (380nm)	2.05%	*Appli. Catal. B: Environ. and Energy* 366 (2025) 125062
27	B[f]QCOF-1	H_2O/O_2	7	9020	8.9% (450nm)	0.23%	*Nat. Commun.* 16, 3493 (2025).
28	TFBP-DHBD COF	H_2O/O_2	7	4300	-	-	*Adv. Mater.* 2025, 37, 2502990.
29	HITMS-COF-21	H_2O/O_2	7	310	2.12% (420nm)	-	*ACS Catal.* 2025, 15, 5683−5693.
30	BBT-ACN COF-1	H_2O/O_2	7	2500	3.19% (500nm)	-	*Adv. Funct. Mater.* 2025, 2424035.
31	TCP-COF	H_2O/O_2	7	3080	0.176% (400nm)	0.04%	*Small* 2025, 21, 2500573.

Section S4. Supporting References

1. Y. Shiraishi, T. Takii, T. Hagi, S. Mori, Y. Kofuji, Y. Kitagawa, S. Tanaka, S. Ichikawa and T. Hirai, *Nat. Mater.*, 2019, **18**, 985–993.
2. Y.-X. Ye, J. Pan, Y. Shen, M. Shen, H. Yan, J. He, X. Yang, F. Zhu, J. Xu, J. He and G. Ouyang, *Proc. Natl. Aca. Sci.*, 2021, **118**, e2115666118.
3. R. Liu, Y. Chen, H. Yu, M. Položij, Y. Guo, T. C. Sum, T. Heine and D. Jiang, *Nat. Catal.*, 2024, **7**, 195-206.
4. A. Kerschbaumer, D. Wielend, E. Leeb, C. Schimanofsky, N. Kleinbruckner, H. Neugebauer, M. Irimia-Vladu and N. S. Sariciftci, *Catal. Sci. & Tech.*, 2023, **13**, 834–843.
5. Y. Qian, Y. Han, X. Zhang, G. Yang, G. Zhang and H. L. Jiang, *Nat. Commun.*, 2023, **14**, 3083.
6. V. Wang, N. Xu, J.-C. Liu, G. Tang and W.-T. Geng, *Comput. Phys. Commun.*, 2021, **267**.
7. X. Zhang, S. Feng, X. Gu, M. Zhou, H. Wang, J. Hua., *Appli. Catal. B: Environ.*, 2024, **366**, 125013.
8. E. Vitaku, C. N. Gannett, K. L. Carpenter, L. Shen, H. D. Abruña and W. R. Dichtel, *J. Am. Chem. Soc.*, 2019, **142**, 16–20.
